# The mechanisms of action of mitochondrial targeting agents in cancer: inhibiting oxidative phosphorylation and inducing apoptosis

**DOI:** 10.3389/fphar.2023.1243613

**Published:** 2023-10-25

**Authors:** Yi Yang, Yahui An, Mingli Ren, Haijiao Wang, Jing Bai, Wenli Du, Dezhi Kong

**Affiliations:** ^1^ Department of Pharmacy, Fourth Hospital of Hebei Medical University, Shijiazhuang, China; ^2^ Institute of Chinese Integrative Medicine, Hebei Medical University, Shijiazhuang, China

**Keywords:** mitochondria targeting drug, mechanism, reactive oxygen species, oxidative phoshorylation, electron transport chain (ETC), mitocans

## Abstract

The tumor microenvironment affects the structure and metabolic function of mitochondria in tumor cells. This process involves changes in metabolic activity, an increase in the amount of reactive oxygen species (ROS) in tumor cells compared to normal cells, the production of more intracellular free radicals, and the activation of oxidative pathways. From a practical perspective, it is advantageous to develop drugs that target mitochondria for the treatment of malignant tumors. Such drugs can enhance the selectivity of treatments for specific cell groups, minimize toxic effects on normal tissues, and improve combinational treatments. Mitochondrial targeting agents typically rely on small molecule medications (such as synthetic small molecules agents, active ingredients of plants, mitochondrial inhibitors or autophagy inhibitors, and others), modified mitochondrial delivery system agents (such as lipophilic cation modification or combining other molecules to form targeted mitochondrial agents), and a few mitochondrial complex inhibitors. This article will review these compounds in three main areas: oxidative phosphorylation (OXPHOS), changes in ROS levels, and endogenous oxidative and apoptotic processes.

## 1 Introduction

Cancer is the second leading cause to death (Bedi et al., 2022) after cardiovascular disease, and it is expected that the number of new cancer patients will reach 20 million worldwide by 2025, which seriously endangers human health, and social and economic development. Mitochondria, being a primary target for most tumors, have become the focus of current research on anticancer drugs. Many signaling and metabolic pathways in cancer cells are changed, which helps cancer develop and progression. Because of altered mitochondrial metabolism and membrane potential, cancer cells are easily affected by therapy that targets mitochondria (Fialova et al., 2021). These drugs, known as “Mitocans,” are demonstrating significant potential (Neuzil et al., 2013). Mitocans have shown a great potential for effectively suppressing a wide range of malignant diseases in a targeted and selective manner (Rohlena et al., 2011).

Instead of normal mitochondrial oxygen consumption (Gatenby and Gillies, 2008; Bedi et al., 2022), tumor cells tend to use glycolysis even in the presence of sufficient oxygen. The Warburg effect, which is the working mode of mitochondria in cancer cells, is shifted to adapt to the altered tumor microenvironment (TME). Upregulated glycolysis is found in well-oxygenated tumor cells to increase glucose consumption and lactate production. Cancer cells undergo a metabolic shift from ATP generation through OXPHOS to ATP generation through glycolysis, even when there is still enough oxygen present in the tumor microenvironment (known as the Warburg effect). This metabolic change leads to many transformed cells relying heavily on aerobic glycolysis for energy, which is faster than OXPHOS but less efficient in terms of ATP production per unit of glucose consumed. Consequently, there is an abnormal increase in the rate of glucose uptake. In addition, circulating cancer cells (CCCs), also known as circulating tumor cells (CTCs), exhibit a significant increase in transcript levels associated with OXPHOS (LeBleu et al., 2014). These CCCs undergo a characteristic EMT phenotype and transition to a bioenergetic program that involves mitochondrial biogenesis and OXPHOS (Sancho et al., 2016). The upregulation of oxidative phosphorylation (OXPHOS) in tumor cells compared to normal cells is well recognized (Ashton et al., 2018; Greene et al., 2022). However, recent studies showed that OXPHOS could be dysregulated (Hernández-Reséndiz et al., 2019; Greene et al., 2022) in some cancers and active during tumor formation and progression (Krasnov et al., 2013). Thus, the process of OXPHOS could be a new breakthrough point for cancer therapy. The heavy dependence of cancer cells on OXPHOS for energy production, cell survival and chemosensitivity, suggests that selective inhibitors of OXPHOS could be used to inhibit tumor growth and prevent or delay drug resistance (Cheng et al., 2020).

Reactive Oxygen Species (ROS) are the products of the body’s normal aerobic metabolism. They are a general term for a class of substances that are composed of oxygen, and are active in nature. They include superoxide anion (O_2·_^−^), hydrogen peroxide (H_2_O_2_), hydroxyl radical (OH^−^), ozone (O_3_), and singlet oxygen (1O_2_), etc., (Arfin et al., 2021).These substances have high chemical reactivity due to the presence of unpaired electrons (Nogueira et al., 2008). The respiratory chain in the inner mitochondrial membrane, where a portion of oxygen is reduced to superoxide radical anion by electron transport chain complexes, is one of the main sources of ROS within the organism. Superoxide radical anion and hydrogen peroxide *in vivo* are mainly generated in the mitochondrial electron transport chain during the transition from state III to IV (Samartsev et al., 2020). The adequate O_2_ environment of the mitochondria allows the highly reduced state of the respiratory chain to have electrons leaked from the substrate end of the respiratory chain and the oxygen end and given to O_2_, which is reduced to O_2·_^−^. Mitochondria are the main source of cellular ROS production, and Complex I and Complex III (CI and CIII) are the main sites of their production. Complex I (NADH) receive two electrons from NADH in the respiratory chain, and then passes them to coenzyme Q (CoQ) through iron-sulfur protein. Complex II (succinate dehydrogenase) obtains electrons from succinate and transfers them to CoQ as well. CoQ then transfers the electrons it received from these two complexes to complex III (coenzyme Q), and ultimately to oxygen (Haapanen and Sharma, 2018). Oxygen molecules capture high-energy electrons at the flavin mononucleotide (FMN) of complex 1, leading to the formation of superoxide radicals. The generation rate of superoxide by flavin in complex 1 depends on the equilibrium between NADH and NAD^+^ (Sarewicz and Osyczka, 2015). Complex II is responsible for transporting electrons produced by the Krebs cycle to the respiratory chain in healthy cells (Barlow and Chance, 1976). Succinate dehydrogenase activity (SDH) plays a crucial role in the Krebs cycle by oxidizing succinate to produce fumarate. This process allows SDHA to supply electrons to complex II. These electrons then transfer to the iron-sulfur core of the SDHB subunit, and ultimately to the succinate coenzyme q oxidoreductase (SQR), which is responsible for reducing CoQ. Complex II also includes SDHC, a subunit that not only produces the resulting polypeptide, but also contains both the active site and the binding site for Heme and CoQ (Ishii et al., 2005). When the SDHC gene is mutated, this alteration will lead to a reduction in complex II activity. Consequently, there will be an excess leakage of electrons from the electron transport process due to a decrease in the affinity between complex II and CoQ. In turn, will result in the uncoupling of electron transfer. In pathological conditions, such as when exposed to pro-apoptotic compounds like anticancer drugs, Factor-related Apoptosis ligand (FasL), or tumor necrosis factor (TNF), the presence of these compounds leads to acidification in the cytoplasm (pH-c) and mitochondria (pH-M). These pH variations cause the SDHA/B subunit to separate from complex II (Ishii et al., 2005), which ultimately reduces SQR activity but does not affect the SDH response. This specific inhibition causes complex II uncoupling, leading to superoxide production and apoptosis. Therefore, complex Ⅱ with intact structure, does not generate a significant amount of ROS. Instead, the impaired electron transport in SDH under pathological conditions is responsible for the production of a large amount of ROS (Yankovskaya et al., 2003). This impairment affects the triphosphopyridine nucleotide (NADPH) level through Krebs cycle dysfunction, which serves as the source and cause of various pathologies. Glutathione (GSH) and glutathione-oxidized (GSSG) regulate oxoglutarate dehydrogenase (OGDH) to produce ROS through S-glutathionylation of different subunits (Mailloux et al., 2016). GSH can amplify ROS formation in mitochondria rather than inhibit it. GSSG at mM concentration can weakly react with OGDH and induce S-glutathionylation on the E2 subunit of OGDH, thus slightly inhibiting the formation of O_2·_^−^/H_2_O_2_ (Gallogly and Mieyal, 2007), and reducing OGDH activity, affecting the whole process of ROS formation. The accumulation of ectopic ROS promotes cell proliferation and malignant transformation (Wang Y. et al., 2021). Additionally, excessive ROS levels disrupt cellular components including chromosomes, proteins and phospholipid bilayers. Increased ROS levels can also activate anti-tumor signaling, leading to oxidative stress-induced cancer cell death (Moloney and Cotter, 2018). The targeted scavenging of mitochondrial superoxide using Mito-TEMPO has been found to effectively inhibit tumor cell migration and prevent spontaneous tumor metastasis in both murine and human tumor models (Porporato et al., 2014). Specific modifications to ROS production and antioxidant defenses have been identified as targets for cancer therapy.

## 2 Targeted therapies on oxidative phosphorylation (OXPHOS)

### 2.1 Targeting mitochondrial complexes

The mammalian mitochondrial respiratory chain is mainly composed of 5 complexes: NADH-ubiquinone oxidoreductase (CI), succinic acid-ubiquinone oxidoreductase (CII), cytochrome Bc1 complex (CIII), cytochrome C oxidase (CIV), and adenosine triphosphate synthase (CV) (Gu et al., 2016; Sousa et al., 2018b). The survival dependence of cancer cells on oxidative phosphorylation provides a therapeutic window for inhibitors of mitochondrial respiration to mitigate tumor growth and treatment resistance (Molina et al., 2018).

Oxidative phosphorylation takes place within the mitochondria of both animal and plant tissues. It is a tightly linked process that involves the oxidation of substrates and the subsequent production of adenosine triphosphate (ATP) (Demirel and Sandler, 2002). During the Kreb’s cycle, hydrogen ions (or electrons) are transported by carrier molecules, namely, NAD or FAD, to the electron transport pumps.

**FIGURE 1:**
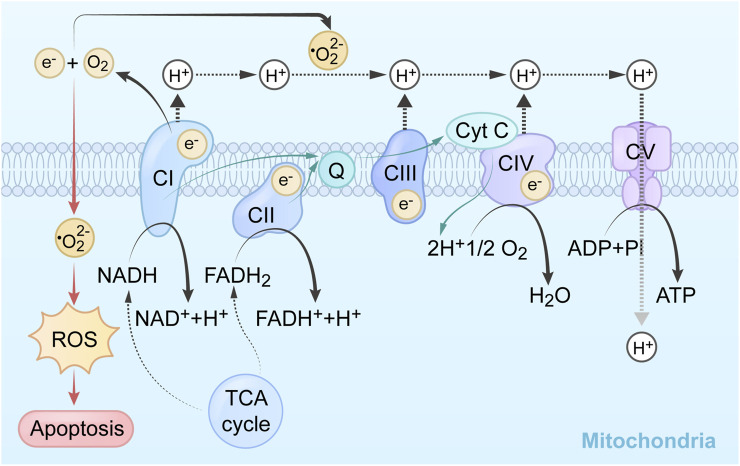
Summary of the mechanism and related pathways of mitochondrial targeting agents.

Reductive substances are oxidized in the inner mitochondrial membrane, generating an H^+^ gradient through the electron transport chain, creating a potential energy difference to catalyze phosphorylation. Complex I in the, ETC is called nicotinamide adenine dinucleotide (NADH) dehydrogenase. It oxidizes NADH to NAD^+^ and transfers an electron pair from NADH to ubiquinone (Q). Flavin mononucleotide, derived from riboflavin (vitamin B2), helps shuttle the electron pair into NADH dehydrogenase. The electron pair is then transferred between iron-sulfur clusters until it reaches ubiquinone. Ubiquinone is reduced to ubiquinol and moves to complex II. This electron transfer releases energy, which is used by the complex to pump 4 H^+^ ions into the intermembrane space. The products of this reaction are NAD^+^, ubiquinol, and 4 H^+^ ions in the intermembrane space (Deshpande and Mohiuddin, 2020). CII is responsible for the oxidation of succinic acid to ferredoxin in the tricarboxylic acid cycle, each takes an H^+^ to form a double bond, then forming a proton gradient and lipid soluble CoQ passing to CIII. CoQ is oxidized to generate 4 H^+^ at CIII through cytochrome B1 and cytochrome c. The intermembrane pumps proton to the last step at CIV through cytochrome a1, and a3 add oxygen to generate water. High concentrations of H^+^ flow from the intermembrane lumen to the mitochondrial matrix through the hydrogen ion channel, pushing the protein rotation to produce conformational changes. The α and β of the protein can connect adenosine diphosphate (ADP) and phosphoric acid (Pi) when loosely bound, and synthesize ATP through the δ subunit on the channel protein add phosphate (Vu Huu et al., 2022). The mechanism of mitochondrial complex action on tumor cells and its application are shown in Table 1, and the mechanisms which shows electron transport chain complex and the relationship between the TCA cycle and OXPHOS are presented in Figure 1.

**TABLE 1:** Sites of action of agents targeting mitochondrial complexes and conclusions.

Drugs	Effect target	Application	Reference
Mitomycin metformin	CI	PANC-1、TNBC	Cheng et al. (2019b)
Mito-DFO	CI	MCF-7、MDA-MB-231	Sandoval-Acuna et al. (2021)
BAY87-2243	CI	-	Sica et al. (2019)
IAC-S010759 (OPi)	CI	Multiple MAPKi-resistant BRAF-mutant melanoma models, acute myeloid leukemia (AML) models	Molina et al. (2018), Vashisht Gopal et al. (2019)
EVT-701	CI	*In vitro* and *in vivo* efficacy of OXPHOS-Eµ-Myc lymphoma in mouse, NSCLC, and NH-B-cell lymphoma models	Luna Yolba et al. (2021)
ME-143/ME-344	CI and mildly inhibit CIII	HEK293T human embryonic kidney	Lim et al. (2015)
Mito-MGN	CI	B16-F10、B16-F0	Cheng et al. (2020), AbuEid et al. (2021)
Lippia organoids extract	CI	MDA-MB-231	Raman et al. (2017), Raman et al. (2018)
Mitochondria-targeted hydroxyurea (Mito-Hu)	CI	Miapaca2	Cheng et al. (2021)
Mito-lonidamine (Mito-LND)	CI	A549、H2030BrM3	Cheng et al. (2019a)
Mito-Tamoxifen	Inhibit CI and CII	HCT116、DU 145、MCF-7	Rohlenova et al. (2017), Ezrova et al. (2021)
α-TOS	CII	MCF-7、MDA-MB-453、NSC、B9rec、B10、B1	Dong et al. (2008)
γ-Tocotrienol (γ-T3)	CII	SGC-7901、MGC-803	Wang et al. (2019)
Mito-VES	CII	human T lymphoma Jurkat, Bax-Jurkat, and Bax-/Bak-Jurkat cells; human mesothelioma	Dong et al. (2011), Liang et al. (2021)
		cells Meso2, Ist-Mes-1, Ist-Mes-2, and MM-BI; human breast cancer	
		cells MCF7 (erbB2-low) and MDA-MB-453 (erbB2-high) and MCF7DD9 cells with transcriptionally inactive p53; human colorectal cells HCT116; human neuroblastoma TetN21 cells; human non-small-cell lung carcinoma cells H1299; human cervical cancer cells HeLa; mouse mesothelioma cells AE17; human nonmalignant mesothelial cells Met-5A; human fibroblasts A014578; rat ventricular myocyte-like cells HL1; and mouse atrial myocyte-like cells H9c2	
GinsenosideRh2	CI, III and V	Hela、C33A、End1/e6e7 Cells	Liu et al. (2021b)
Mito-Atovaquone	CI, CIII	LKR13、unscc680、LKR13-luc	Mudassar et al. (2020), Huang et al. (2022)
Capsaicin	CI, CIII	BxPC-3、AsPC-1	Pramanik et al. (2011)
Mitochondria-targeted carboxy-proxyl (Mito-CP)	COX IV、Mcl-1 were dramatically downregulated	TT、MZ-CRC-1	Starenki and Park (2013), Cheng et al. (2015), Hong et al. (2017)
Mito-CP-Ac	CIII	MiaPaCa-2、 PANC-1、 MCF-7、 MDA-MB-231、 MCF-10A and A431 Cells	Zhou et al. (2022)

OXPHOS of mitochondria is a crucial regulator of tumor proliferation. The amino acids arginine 21 (R21) and lysine 108 (K108) of mitochondrial ribosomal protein S23 (MRPS23) are methylated by the enzymes protein arginine methyltransferase 7 (PRMT7) and set domain-containing protein 6 (SETD6) (Liu L. et al., 2021). Degradation of MRPS23 increases mtROS levels and suppresses OXPHOS, encouraging the invasion and metastasis of breast cancer cells. With the goal to maintain an ongoing supply of energetic substrates (Martínez Winkler and Smith, 2021) and ensure adequate energy for tumor spreading and migration, the metabolic shift toward higher OXPHOS during spreading and migration requires physical regulation of cytoskeleton remodeling (Urra et al., 2021).

TPP^+^ is a compound consisting of three benzene rings that can bind to various functional molecules, resulting in the formation of positively charged particles that can penetrate the hydrophobic membrane of mitochondria (Lin et al., 2021). Many biologically active substances have been combined with TPP^+^ to specifically target mitochondria. The modification of TPP^+^ has been widely employed in cancer treatment due to its enhanced targeting capabilities. For instance, the chemical conjugation of doxorubicin (Dox) with TPP^+^ has demonstrated excellent mitochondrial targeting efficacy, leading to tumor cell apoptosis via the mitochondrial pathway and overcoming drug resistance (Kalinovich et al., 2016). These modified nanocarriers have exhibited remarkable mitochondrial targeting abilities and demonstrated significant anti-tumor effects both in laboratory experiments and animal studies (Wang H. et al., 2021).

#### 2.1.1 Inhibitors of mitochondrial complex I

Metformin, a CI inhibitor, effectively disrupts cellular and mitochondrial respiration by inhibiting complex I, which is closely associated with NADH-related respiration (Pollak, 2012). Furthermore, it exerts its anti-proliferative effects by activating AMPK and inhibiting mTOR, thereby facilitating a high level of ROS and reducing mitochondrial respiration and ATP production to inhibit tumor development (Morales and Morris, 2015). In contrast to rotenone, the accumulation of metformin in mitochondria is a reversible process (Bridges et al., 2014) and can be used in combination with other drugs (Andrzejewski et al., 2014). Analogs of Met (Mito-Met) conjugated to varying alkyl chain lengths containing a triphenylphosphonium cation (TPP^+^) as a stand-alone antitumor agent (Cheng et al., 2019b), produced synergistic antiproliferative effects in combination with iron chelators and deferoxamine (DFO). These effects have been demonstrated in pancreatic cancer cells and triple-negative breast cancer (TNBC) cells. Mito-DFO (Sandoval-Acuna et al., 2021), which was modified the classical iron chelator deferoxamine (DFO) by tagging it with the TPP^+^, inhibits mitochondrial respiration by inhibiting CI, and leads to the production of ROS substances, fragmentation of the mitochondrial network and the induction of mitochondrial autophagy. In addition, impaired iron-sulfur [Fe-S] cluster/hemoglobin biosynthesis causes destabilization and loss of activity of [Fe-S] cluster/hemoglobin containing enzymes, both of which together inhibit tumor cell proliferation and invasion.

BAY87-2243 (B87) a CI Inhibitor, can be coupled to the metabolic activity of dimethyl α-ketoglutarate (DMKG) by inhibiting the respiratory chain. Dimethyla-ketoglutarate (DMKG) is a non-toxic compound that serves as a cell-permeable precursor of a-ketoglutarate. Interestingly, when combined with B87 or other OXPHOS inhibitors, DMKG demonstrates the ability to effectively eliminate cancer cells. The cytotoxicity induced by the combination of B87 and DMKG was reduced by the addition of dimethyl succinate, the complex II substrate succinate, and the knockdown of isocitrate dehydrogenase 1 (IDH1). The inhibition of the respiratory chain, specifically the succinate bypass, is linked to the metabolic activity of DMKG. This metabolic activity may involve the (reverse) conversion of IDH1 to isocitrate in the cytoplasm. This mechanism helps to explain the synergistic cytotoxic effect observed when combining B87 with DMKG. It has been verified in breast cancer MCF7 cells, human colorectal cancer HT-29 cells, glioma H4 cells, osteosarcoma U-2 OS cells, H460 and HCT116 cells (Sica et al., 2019). Furthermore, treatment with DMKG resulted in an increase in TCA intermediates, glucose, oligosaccharide, and 2-Deoxy-D-glucose (2-DG) levels. Interestingly, DMKG alone did not hinder mitochondrial respiration but rather enhanced oxygen consumption in HCT116 and H460 cells. Notably, the combination of respiratory chain inhibition and DMKG exhibited a robust suppression of glycolysis, leading to a lethal bioenergetic crisis, ultimately accomplishing the objective of eliminating tumor cells. The mousedouble minute 2 (MDM2)-dependent, tumor suppressor protein p53 (TP53) non-dependent transcriptional reprogramming and alternative exon usage affecting multiple glycolytic enzymes, completely blocking glycolysis (Sica et al., 2019) and improving anti-tumor capacity *in vitro* and *in vivo*. Simultaneous inhibition of OXPHOS and glycolysis triggers bioenergetic catastrophe and ultimately leads to apoptosis, a program involving disruption of the mitochondrial network and activation of poly (ADP-ribose) polymerase 1(PARP1), apoptosis-inducing factor 1 (AIFM1) and apyrimidinic endodeoxyribonuclease 1 (APEX1). These results indicate that targeting metabolic change can be used to develop therapeutic options, but were not promoted to the clinic because of high toxicity.

IAC-S010759(OPi) has same functional backbone but has been attenuated conduction (Molina et al., 2018; Vashisht Gopal et al., 2019), a directed CI inhibitor, was shown to significantly inhibit the growth of melanoma models with multiple MAPKi resistant BRAF mutation *in vivo* at tolerated doses, and strongly inhibited proliferation and induced apoptosis in OXPHOS-dependent brain cancer and acute myeloid leukemia (AML) models. Through OPi treatment, the intracellular steady state levels of CI substrate NADH as to nucleotide monophosphate (NMP) were enhanced, while the levels of nucleotide triphosphate (NTP) were reduced, which was the result of CI inhibition. OPi promoted the entry of glucose into glycolysis, inhibited the entry of glucose and glutamine into the TCA cycle, and reduced the cellular nucleotide and amino acid pools, using asparagine as a rate-limiting marker to inhibit the process of OXPHOS.

EVT-701 is a novel, potent and selective CI inhibitor with an original chemical scaffold designed to circumvent the side effects of B87 and Opi and to act as a hypoxia-inducible factor (HIF)-1α-destabilizer (Luna Yolba et al., 2021). EVT-701 has demonstrated a dose-dependent inhibition of 80% of NADH oxidation and ADP phosphorylation to ATP, and has the potential to become an anticancer agent specifically targeting OXPHOS-dependent tumors, and has been shown to prolong survival in mice bearing OXPHOS-Eµ-Myc lymphomas, but not in glycolytic- Eµ- Myc-tumor-bearing mice. Contrary to metformin, which was activated by adenosine 5‘-monophosphate-activated protein kinase (AMPK), EVT-701 is driven by Liver kinase B1 (LKB1) status, making EVT-701 suitable as a combination therapy to counteract tumor metabolic changes. The preclinical validation (Luna Yolba et al., 2021) already shown *ex vivo* efficacy in non-small cell lung cancer (NSCLC) cells and NH-B cell lymphoma models.

In the study of isoflavone analogues ME-143 and ME-344 (Lim et al., 2015), it was found that direct inhibition of CI by ME-344 and mild inhibition of CIII can decrease mitochondrial respiration produced ROS-induced phosphorylation of mitochondrial extracellular regulated protein kinases (ERK), and cause translocation of Bax to the outer mitochondrial membrane to alter mitochondrial permeability. Then activated several signaling pathways after release of pro-apoptotic molecules, thus achieving the expectation of selective induction of apoptosis in cancer cells.

Mito-magnolol (Mito- MGN) (Cheng et al., 2020; AbuEid et al., 2021) is a mitochondria-targeted chondroitin analogue. Mito- MGN significantly and dose-dependently reduces oxygen consumption rate (OCR) in B16-F10 cells and B16-F0 cells, and the reduction in OCR and oxidative metabolism downregulates protein kinase B (AKT) activity and AktFOXO1 levels. The immune T cells are activated both in Akt/FOXO signaling and cell cycle progression, which is expected to bring greater benefit and less toxic effects in clinical application.

The novel L. origanoides extract (LOE) (Raman et al., 2018) regulated mitochondrial OXPHOS primarily by vivo and *ex vivo*, which reduces CI oxygen consumption and cell proliferation, blocking inhibiting the expression of several subunits of CI. LOE inhibits cell metabolism and reduces the expression of all core subunits of CI by down-regulating key TCA cycle enzymes, mitochondrial lipid, and amino acid metabolic pathways, as well as CIV subunits. Multiple, ETC complexes were significantly downregulated in MDA-MB-231 cells. LOE inhibits NF-κB signaling by reducing RIP1 protein levels in MDA-MB-231 cells (Raman et al., 2017). NF-κB signaling has been shown to facilitate enhanced inflammatory status and survival of TNBC cells.

Mitochondria-targeted hydroxyurea (Mito-Hu) (Cheng et al., 2021) adopts of a strategy that uses triphenyl-phosphonium ions on alkyl groups of different chain lengths to replace the hydroxyl group in HU hydroxyurea, and after increasing the hydrophobicity of Mito-Hu. Its mechanism is to reduce oxygen consumption of CI and CIII, while inhibiting neutrophil and T cell responses. Silencing the S100A4 and NDUFS2 genes can inhibit CI activity (Boddapati et al., 2008), upregulate hexokinase expression, which shifts metabolism to glycolysis, decreases cellular ATP levels, and significantly reduces tumor metastasis and growth *in vivo*.

Mito-lonidamine (Mito-LND) (Cheng et al., 2019a) inhibits lung tumor progression and brain metastasis through a combination of mechanisms including inhibition of mitochondrial bioenergetics, stimulation of ROS formation, oxidation of mitochondrial peroxiredoxins, inactivation of AKT/mTOR/p70S6K signaling pathway and induction of autophagic cell death in lung cancer cells. Mito-LND inhibits CI and CII activity, and Mito-LND-induced H_2_O_2_ production can override the ability of mitochondria to degrade peroxides.

Targeting tamoxifen (Mito-Tam) (Rohlenova et al., 2017), a modified formulation, has been validated as a new strategy for the treatment of Her-2high breast cancer, where the genetic phenotype re-organizes the, ETC so that Mito-Tam is more sensitive to it. Both *in vitro* and *in vivo* experiments have demonstrated that inhibition of CI and disruption of the respiratory super complex in the Her2-positive background, leads to increased production of ROS material and cell death. By overcoming drug resistance (Ezrova et al., 2021) in patients with SMAD family member 4 gene (SMAD4)-deficient or Transforming Growth Factor-β (TGF-β) signaling-mediated pancreatic cancer, Mito-Tam contributes to the development of a rational predictive marker for mitochondrial-targeted therapy in pancreatic cancer patients with SMAD4 expression. Currently, Zuzana Bielcikova et al. have conducted a phase I clinical study on Mito-Tam to assess its safety, determine the maximum tolerated dose, establish the appropriate dose for phase Ⅰb, and identify potential target groups for subsequent malignant tumor treatment (Bielcikova et al., 2023). Notably, Renalcellcarcinoma (RCC) patients with metastatic disease showed significant improvement after 3-4 treatment cycles with Mito-Tam.

#### 2.1.2 Inhibitors of mitochondrial complex Ⅱ

Alpha-vitamin E succinate (α-TOS), an essential vitamin E analogue, stands out as a redox-silent and semisynthetic compound. It is derived by replacing the hydroxyl group on the chroman head of α-tocopherol with a succinyl group. Notably, α-TOS possesses remarkable antineoplastic properties (Angulo-Molina et al., 2014). Alpha-vitamin E succinate (α-TOS) inhibits the SDH activity of CII by interacting with proximal and distal ubiquinone (UbQ) binding sites (Dong et al., 2008), resulting in the recombination of SDH-generated electrons with molecular oxygen to generate ROS. CybL mutant cells with abnormal CII function are unable to accumulate ROS and undergo apoptosis. The recombination of functional CII makes CybL mutant cells susceptible to α-TOS, and functional CII can be used as a new target for tumor cell inhibition.

γ-Tocotrienol (γ-T3) (Wang et al., 2019) is a homologue of tocopherol and γ-T3-induced apoptosis is consistent with γ-T3 decreasing NDUFB8 and SDHB levels leading to overproduction of ROS, impairment of OXPHOS and compensatory elevation of glycolysis. In addition, T3-induced apoptosis in cancer cells is associated with a mitochondria-dependent apoptotic pathway and involves concomitant activation of caspase-3/-9, as with PARP cleavage to induce apoptosis in cancer cells.

Mitochondrially targeted vitamin E succinate (Mito-VES) has high levels of apoptotic activity (Dong et al., 2011) and its anti-tumor mechanism is the rapid production of ROS, with α-TOS acting as a B cell lymphoma 2 (BCL2) homology domain 3 (BH3) mimetic at the molecular level, then effectively sensitizing cancer cells to other drugs and inducing apoptosis by impacting CII. VE analogues also interfere with the function of UbQ as a natural acceptor for the electrons generated by the succinate dehydrogenase activity of CII during the conversion of succinate to ferredoxin, which reduces ATP production and decrease normal mitochondrial respiratory metabolism. The combination of Mito-VES and doxorubicin hydrochloride (Liang et al., 2021) showed significantly enhanced antitumor efficacy in double-loaded nanovesicles in nude mice bearing xenograft-resistant human chronic granulocytic leukaemia K562/ADR tumors, with a tumor inhibition rate of 82.38%. Overall, this study provides a safe, and promising strategy for precision drug delivery to improve cancer treatment.

#### 2.1.3 Inhibitors of mitochondrial complex III, IV, and Ⅴ

In mammals, mitochondrial complex III serves as an essential electron carrier. It accepts electrons from CoQ and transfers them to complex IV. Inhibition of the mitochondrial electron transport chain (ETC.), in conjunction with targeted therapy, has demonstrated anti-tumor effects. The oxidation of mitochondrial ubiquinone, which occurs when panthenol is converted to ubiquinone, is necessary for the development of tumors (Martínez-Reyes et al., 2020). This oxidation process is essential for the subsequent induction of oxidative TCA cycle and dihydroorotate dehydrogenase (DHODH) activity. The cytochrome C oxidase complex is found in mitochondrial complex IV. It feeds oxygen with electrons. The last stage of electron transport is catalyzed by mitochondrial complex IV, which has 14 protein subunits and a number of cofactors. In brief, electrons from cytochrome C in CIV are transferred from CuA to cytochrome α, and then to the oxygen reaction center composed of cytochrome α3 and CuB. The energy released during electron transfer alters the CIV protein structure. Half of the protons in the mitochondrial matrix were directly pumped into the mitochondrial membrane space via the H^+^ channel, while the other portion was transported to the oxygen reaction center through the K and D channels (Ghane et al., 2018) to reduce oxygen and produce water. Mitochondrial complex V, often known as ATP synthase, is made up of four complex respiratory chain enzymes that work together to produce ATP. Complex V is a rotatable enzyme which is using a proton electrochemical potential gradient on both sides of the mitochondrial inner membrane during electron transport to produce ATP (Sousa et al., 2018a).

Research on ginsenoside Rh2 (G-Rh2) (Liu Y. et al., 2021) showed that G-Rh2 significantly inhibited the activity of ETC complexes I, III and V by targeting CIII to induce ROS production, inhibit mitochondrial OXPHOS and glycolysis and decrease mitochondrial transmembrane potential, thereby inducing cell apoptosis. In this study, the results of molecular docking revealed that CIII exhibited a greater affinity with Rh2. Hence, it can be inferred that the inhibition of CI and CIV was a consequence of the indirect effect of CIII. Previous reports have established a close connection between CI and CIII through protein subunit interactions, which could potentially explain the decreased activity of CI influenced by CIII (Gu et al., 2016).

Anti-parasitic drugs (Mudassar et al., 2020) (atovaquone, ivermectin, clonidine, mefloquine and quinacrine) are effective against cancer through mechanisms such as inhibition of mitochondrial metabolism and tumor hypoxia, as well as induction of DNA damage, and can be used in combination with radiotherapy to achieve highly effective results. Hypoxia is important in solid tumors, including high grade gliomas (HGGs), as it increases HIFs that help cancer cells survive in low oxygen conditions (Zheng, 2012). HIFs also drive cancer cells to use glycolysis, then using anti-parasitic drugs to upregulate hypoxia-inducible factors (HIFs) as a potential strategy to inhibit tumors. Atovaquone, as a well-known anti-protozoal drug, can inhibit CI and CIII activity in cancer cells through modified Mitochondria-targeted atovaquone (Mito-ATO) (Huang et al., 2022), inhibit mitochondrial electron transport, OXPHOS and glycolysis multiple pathways, induce apoptosis, and inhibition of CIII triggers tumor immunosuppressive function. An enhancement of tumor-infiltrating CD4^+^ T cells was observed in Mito-ATO-treated cells and increased the anti-tumor activity of Programmed Death 1(PD-1) blockade immunotherapy.

The mechanism of capsaicin-mediated apoptosis in pancreatic cancer cells was primarily through the inhibition of CI and CIII enzymes thereby inducing ROS production, which inhibited BxPC-3 and AsPC-1 cell development from both ROS and ATP production perspectives. Since both catalase and EUK-134 significantly prevented the release of cytochrome c into the cytoplasm and capsaicin-mediated cleavage of caspase-3 (Pramanik et al., 2011), inhibition of apoptosis by antioxidants due to altered OXPHOS processes leading to excessive ROS production is a proven anti-tumor strategy.

Mitochondria-targeted carboxy-proxyl (Mito-CP) (Starenki and Park, 2013) is a mitochondria-targeted redox-sensitive agent with medullary thyroid carcinoma (MTC) tumor suppressive properties. Mito-CP caused cytotoxicity mainly by inhibiting the mitochondrial activity critical to maintain bioenergetic and REDOX equilibrium, however the mitochondria-specific delivery of the CP fragment of Mito-CP is essential for tumor inhibition. In addition, Mito-CP exhibited a stronger ability to induce laminin A fragmentation in the melanoma system, which serves as a crucial indicator of caspase-induced apoptosis. The activation of the caspase cascade was found to significantly decrease the expression of cytochrome c oxidase IV (COX IV) and myeloid cell leukemia-1 (Mcl-1) (Hong et al., 2017), while having no impact on Bcl-2 levels. By perturbing superoxide. Mito-CP lowers ROS activity in the tumor microenvironment. Conversely, it led to an upregulation of Bcl-xL levels. Mito-CP decreased ROS activity in the tumor microenvironment by disproportioning superoxide. Conversely, it led to an upregulation of Bcl-xL levels. Furthermore, Mito-CP effectively reduced ROS activity in the tumor microenvironment by disrupting the balance of superoxide. For inhibiting the proliferation of pancreatic cancer cells, Mito-CP and a synthetic cationic acetamide analog (Mito-CP-Ac) suppressed cellular energy metabolism function (Cheng et al., 2015), activated AMPK energy-sensing pathway, and changed the roles of glycolysis and citrate in mitochondrial bioenergy metabolism.

Mitoquinone (Mito-Q) is a specialized antioxidant that specifically targets mitochondria to effectively eliminate excessive ROS. It is composed of TPP^+^ and the ubiquinone portion of CoQ (Fujimoto and Yamasoba, 2019). Mito-Q induces mitochondrial uncoupling (Fedorenko et al., 2012a; Demine et al., 2019), as manifested by reduced ATP, reduced mitochondrial membrane potential (MMP) and increased mitochondrial energy production. Uncoupling protein 2(UCP2) is a member of the mitochondrial anion transporter superfamily (SLC25) and is located in the inner mitochondrial membrane (Nicholls, 2021). Notably, UCP2’s function as a proton transporter renders it a member of the uncoupling protein family. Research indicated that UCP2 deletion in oocytes increased thermogenesis, decreased ATP, and decreased MMP, indicating that UCP2 downregulation could be a factor in Mito-Q-induced mitochondrial uncoupling (Fedorenko et al., 2012b). Specific deletion of UCP2 leads to an enhancement in thermogenesis, while the expression of UCPs is reduced as a result of mitochondrial uncoupling induced by Mito-Q. In the experiment targeting mid-stage oocytes, downregulation of UCP2 expression (Zhou et al., 2022) was used to increase autophagy and induce mitochondrial quiescence, as evidenced by reduced MMP and ROS, reduced lipid metabolism, decreased ATP content and stable thermogenesis, which can be used as a reversible regulatory scheme to maintain the metabolic pattern of the tumor microenvironment.

A switch from oxidative phosphorylation to aerobic glycolysis to create ATP is one of the key modifications that most malignant cancer cells experience (Warburg effect). The combination of mitochondria-targeted drugs (MTD) (drugs such as Mito-CP and Mito-Q) and antiglycolytic agents represented by 2-DG will synergistically enhance the selectivity of cancer cell cytotoxicity compared to administration alone (Cheng et al., 2012). The dual targeting of mitochondrial bioenergy metabolism by glycolytic inhibitors such as MTDs and 2-DG May provide a promising strategy for chemotherapy.

### 2.2 Targeting the by-product ROS of electron transport chain

The production of endogenous ROS primarily originates from two sources: the mitochondrial respiratory chain and NADPH oxidases (NOxs) (Sarniak et al., 2016). Specifically, the mitochondrial respiratory chain generates ROS as a by-product. Additionally, the peroxisome and endoplasmic reticulum membranes are also identified as cellular sites of ROS production.

In the process of oxidative phosphorylation and energy transfer, about 1% of the electrons leak out of the potential site in CI, the Fe-S cluster, to generate superoxide radical anion by one-electron reduction of molecular oxygen, and some are oxidized at CII flavin, and then in CIII (Vercellino and Sazanov, 2022). Superoxide is released to the inner mitochondrial membrane and matrix (Dröse and Brandt, 2012). Additionally, ROS function as signaling molecules in cancer, affecting cellular pathways and causing diseases when there is too much accumulation. It is crucial to maintain a balanced level of ROS for normal cell survival. Excessive ROS accumulation can lead to the malignant transformation of normal cells by disrupting the physiological signaling network and promoting cell proliferation (Sahoo et al., 2022). Excessive levels of ROS destroy cellular components, including proteins, lipid bilayers and chromosomes, leading to cell (Redza-Dutordoir and Averill-Bates, 2016). OGDH is the rate-limiting enzyme of the tricarboxylic acid cycle, which regulates the production of succinate by decarboxylation of α-ketoglutarate (Cui et al., 2021). The fourth step in the tricarboxylic acid cycle is the oxidative decarboxylation of α-ketoglutarate to form succinyl-CoA, which is formed by the alpha-ketoglutarate dehydrogenase complex (A-KGDH). A-KGDH or 2-oxoglutarate dehydrogenase complex (OGDC) catalysis. This is the second oxidative decarboxylation, yielding NADH. KGDH and pyruvate dehydrogenase (PDH) belong to the same family (2-oxoate dehydrogenase complex family), so the catalytic mechanism is very similar to PDH. E1 is α-ketoglutarate dehydrogenase, E2 is dihydrolipoamide succinyl-transferase, and E3 is dihydrolipoamide dehydrogenase. KGDH not only regulates the flow of the tricarboxylic acid cycle, but also is associated with oxidative stress in cells. ROS are generated during KGDH catalysis and are increased in NAD deficiency. ROS can inhibit the activities of cis-aconitase and KGDH (Tretter and Adam-Vizi, 2005), which plays an important role in the regulation of tricarboxylic acid cycle under re-oxidative stress conditions.

Oxidative stress is caused by the production of ROS by cells, which is controlled by cellular antioxidant mechanisms. When the production of ROS surpasses the cell’s antioxidant capacity, it results in damage to cellular components. Several pathways contribute to ROS production, including the mitochondrial electron transport chain, inflammatory signaling, and endoplasmic reticulum stress (Ralph et al., 2010). Certain drugs have the ability to hinder alternative sources of energy metabolites used by cancer mitochondria. This leads to oxidative stress and the generation of ROS. Consequently, this can induce apoptosis in cancer cells, making these drugs potential targets for tumor therapy (Yang et al., 2016a). Mitochondria-targeted antioxidants have the ability to accumulate in mitochondria and effectively reduce oxidative damage.

**FIGURE 2:**
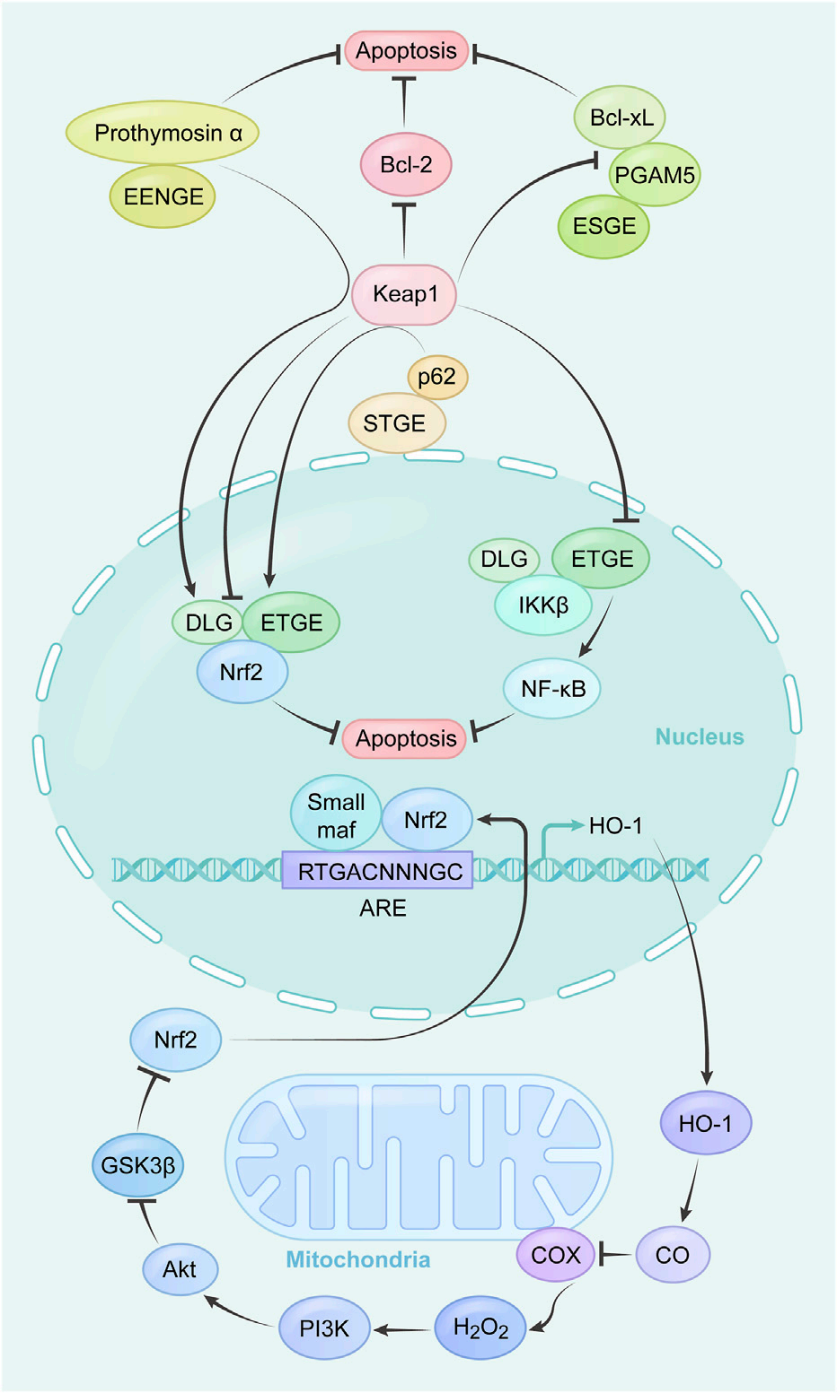
Summary of the mechanism and related apoptosis pathways of mitochondrial targeting agents.

Tumor cells achieve a balance between fine-tuned energy requirements and mitochondrial ROS status through the regulation of mitochondrial respiration to maintain an optimal survival environment, with key players including Bcl-2 and cytochrome c oxidase (COX). Increased COX activity, oxygen consumption and mitochondrial respiration were found in tumor cells overexpressing Bcl-2. Excitingly, Bcl-2 was also able to regulate mitochondrial respiration and COX activity (Chen and Pervaiz, 2007) in the face of increasing levels of ROS triggered by inhibitors of the mitochondrial complex. The mechanisms of related proteins on cytoplasm and nucleus and their pathways are summarized in Table 2, and the combined application experiments are shown in Table 3. And the mechanisms which shows electron transport chain complex and proteins related with apoptosis are presented in Figure 2.

**TABLE 2:** Other mechanisms of mitochondrial targeting agents and applications.

Drugs	Mechanism	Application	Reference
Mito-Q	Induction of mitochondrial uncoupling	MCF-7, MCF-10A, and MDA-MB-231 cells	Cheng et al. (2012)
Lupane Triterpenoid Derivatives	The dose-dependent induction of ROS production reduced the cell membrane potential	K562、A549、ECA-109、HepG2、HL-7702、HL-60	Xu et al. (2022)
3-O-(3′-acetylphenylacetate)-betulin with triphenyl phosphonium	Arrest of the tumor cell at the G2/M phase, caused ROS overproduction, decreased ψM, and induced apoptosis via the mitochondria pathway.	A549、U87、Hela、MDA-MB231、HCT116	Kariyil et al. (2021)
Chloroform Fraction of Methanolic Extract of Seeds of Annona muricata (CMAM)	To induct S Phase arrest and ROS dependent caspase activated mitochondria-mediated apoptosis	MDA-MB-231	Lin et al. (2011)
18b-Glycyrrhetinic acid derivatives	The dose-dependent induction of cell apoptosis	A549、U87、Hela、MDA-MB231、HCT116、NCM460	Jin et al. (2019)
Triphenyl phosphonium conjugated glycyrrhetinic acid derivatives	Apoptosis cells through the mitochondrial pathway via the collapse of mitochondrial membrane potential, reactive oxygen species production and the activation of caspase-9 and caspase-3	HepG-2、A549、MCF-7、HT-29、A2780、HL-7702	Zheng et al. (2014)
Curcumin Derivative B63 (B63)	ROS elevation caused by ER stress and mitochondrial dysfunction	SW620、SW480、HCT116、HIEC	Kim et al. (2020)
CADD522	Regulation of ROS levels induces apoptosis	Hs578t、RUNX2 KD、MCF7-RUNX2	Liu et al. (2018)
HA-ionic-TPP-DOX	Increased ROS production and slightly decreased mitochondrial membrane potential	MCF-7/ADR	Wang et al. (2014a)
Bardoxolone methyl (CDDO-Me)	Apoptosis was induced by increasing ROS and decreasing intracellular glutathione levels	EC109、KYse70	Wang et al. (2015), Ju et al. (2021a)
Triphenyl phosphonium derivatives of CDDO	Mitochondrial membrane potential decreased and cell apoptosis was induced	MCF-7	Szabo et al. (2021)
Mito-chondriotropic PAP-1 Derivatives	Blockade of IMM Kv1.3 resulted in an initial hyperpolarization and apoptosis caused by cytochrome C release after ROS release	Jurkat T Cells、Primary pathology CD19+/CD5+ B Cells, B16F10 Melanoma Cells, Pathological B-CLL Cells	He et al. (2022)
DNA methyltransferase (DNMT) inhibitor RG108	RG108 treatment could reduce ROS accumulation and inhibit apoptosis, which is mediated, at least partially, through LRP1ePI3K/AKT signaling pathway	HEI-OC1	Xu et al. (2018)
Pyruvate Dehydrogenase Kinase (PDK1) Inhibitors	The extracellular acidification rate and lactate formation were decreased, and ROS production was increased	NCI-H1650	Sharma et al. (2019)
Mitochondrial targeted Doxorubicin (Dox) delivery System based on N-(2-hydroxypropyl) methyl acrylamide copolymer and Mitochondrial Distributed Bcl-2 Function switching peptide NuBCP-9 Delivery System (PN9)	Imbalance of mitochondrial homeostasis	4T1	O'Neill et al. (2016)
Azelastine	Inducing ROS levels to increase is helpful to increase oxidative stress and stress in rough endoplasmic reticulum and induce apoptosis	Hela	Bazhin et al. (2016)
SkQ1	Scavenging excess free radicals in mitochondria	Mia-Paca、Dan-G	Yang et al. (2016b)

**TABLE 3:** Research progress and effect of mitochondrial targeting agents in combination with other drugs.

Drugs	Effect target	Application	Drug combination	Reference
Mitomycin metformin	CI	PANC-1、TNBC	Iron chelators, DFX, and deferoxamine synergistically inhibited proliferation	Cheng et al. (2019b)
BAY87-2243	CI	-	Combined with DMKG affects metabolic activity	Sica et al. (2019)
Mito-Tamoxifen	CI, CII	HCT116、DU 145、MCF-7	Doxorubicin-induced apoptosis was attenuated	Rohlenova et al. (2017), Ezrova et al. (2021)
Mito-VES	CII	human T lymphoma Jurkat, Bax-Jurkat, and Bax-/Bak-Jurkat cells; human mesothelioma	The combination of Mito-VES and doxorubicin hydrochloride significantly enhanced the anti-tumor effect of dual-loaded nanocapsules in nude mice bearing xenotransplantation drug resistant human chronic myeloid leukemia K562/ADR tumor, and the tumor inhibition rate was up to 82.38%	Dong et al. (2011), Liang et al. (2021)
		cells Meso2, Ist-Mes-1, Ist-Mes-2, and MM-BI; human breast cancer		
		cells MCF7 (erbB2-low) and MDA-MB-453 (erbB2-high) and MCF7DD9 cells with transcriptionally inactive p53; human colorectal cells HCT116; human neuroblastoma TetN21 cells; human non-small-cell lung carcinoma cells H1299; human cervical cancer cells HeLa; mouse mesothelioma cells AE17; human nonmalignant mesothelial cells Met-5A; human fibroblasts A014578; rat ventricular myocyte-like cells HL1; and mouse atrial myocyte-like cells H9c2		
Mito-CP-Ac	CIII and inhibit mitochondrial oxygen consumption	MiaPaCa-2, PANC-1, MCF-7, MDA-MB-231, MCF-10A and A431 Cells	Combined use of 2-DG would synergistically enhance cytotoxic selectivity in cancer cells	Zhou et al. (2022)
Mito-Q	Induction of mitochondrial uncoupling	MCF-7, MCF-10A, and MDA-MB-231 cells	Combined use of 2-DG would synergistically enhance cytotoxic selectivity in cancer cells	Cheng et al. (2012)

Abbreviations: ROS, reactive oxygen species; OXPHOS, oxidative phosphorylation; TME, tumor microenvironment; ETC., electron transport chain; Complex I, CI; Complex Ⅱ, CⅡ; Complex Ⅲ, CⅢ; Complex Ⅳ, CⅣ; Complex Ⅴ, CⅤ; Succinate dehydrogenase, SDH; succinate dehydrogenase complex subunit A, SDHA; succinate dehydrogenase complex subunit B, SDHB; succinate dehydrogenase complex subunit C, SDHC; succinate coenzyme q oxidoreductase, SQR; coenzyme q,CoQ; Factor-related Apoptosis ligand, FasL; tumor necrosis factor, TNF; acidification in the cytoplasm, pH-c; acidification in the mitochondria, pH-m; triphosphopyridine nucleotide, NADPH; glutathione, GSH; Glutathione-oxidized, GSSG; oxoglutarate dehydrogenase, OGDH; TCA, tricarboxylic acid cycle; adenosine triphosphate, ATP; adenosine diphosphate,ADP; phosphoric acid,Pi; nicotinamide adenine dinucleotide, NADH; The amino acids arginine 21, R21; lysine 108,K108; mitochondrial ribosomal protein S23,MRPS23; the enzymes protein arginine methyltransferase 7,PRMT7; set domain-containing protein 6,SETD6; Mito-Met, metformin; DFO, deferoxamine; TNBC, triple-negative breast cancer; B87, BAY87-2243; DMKG, dimethyl α-ketoglutarate; isocitrate dehydrogenase 1,IDH1; mousedouble minute 2, MDM2; 2-Deoxy-D-glucose, 2-DG; TP-53, tumor suppressor protein p53; poly (ADP-ribose) polymerase 1, PARP1; apoptosis-inducing factor 1, AIFM1; apyrimidinic endodeoxyribonuclease 1, APEX1; OPi, IAC-S010759; AML, acute myeloid leukemia; NMP, nucleotide monophosphate; NTP, nucleotide triphosphate; hypoxia-inducible factor, HIF; adenosine 5′-monophosphate-activated protein kinase, AMPK; Liver kinase B1,LKB1; NSCLC, non-small cell lung cancer; ERK, extracellular regulated protein kinases; Mito- MGN, Mito-magnolol; OCR, oxygen consumption rate; protein kinase B, AKT; The novel L. origanoides extract LOE; Mito-Hu, mitochondria-targeted hydroxyurea; Mito-LND, Mito-lonidamine; Mito-Tam, Targeting tamoxifen; SMAD, family member 4 Gene, SMAD4; Transforming Growth Factor-β, TGF-β; alpha-vitamin E succinate,α-TOS; UbQ, ubiquinone; γ-T3, γ-Tocotrienol; Mitochondrially targeted vitamin E succinate,Mito-VES; B cell lymphoma 2 (BCL2) homology domain 3, BH3; dihydroorotate dehydrogenase, DHODH; ginsenoside Rh2,G-Rh2; high grade gliomas, HGGs; Mitochondria-targeted atovaquone,Mito-ATO; Programmed Death 1, PD-1; mitochondria-targeted carboxy-proxyl,Mito-CP; MTC, medullary thyroid carcinoma; cytochrome c oxidase IV, COX IV; myeloid cell leukemia-1, Mcl-1; a synthetic cationic acetamide analog,Mito-CP-Ac; Mitoquinone,Mito-Q; MMP, mitochondrial membrane potential; Uncoupling protein 2, UCP2; solute carrier family 25, SLC25; mitochondria-targeted drugs,MTD; NOxs, NADPH, oxidases; alpha-ketoglutarate dehydrogenase complex, A-KGDH; 2-oxoglutarate dehydrogenase complex, OGDC; pyruvate dehydrogenase, PDH; COX, cytochrome c oxidase; CMAM, the chloroform fraction; B63, Curcumin Derivative B63; proliferating cell nuclear antigen, PCNA; pipyine,PIP; runt-related transcription factor 2,RUNX2; doxorubicin, DOX; triphenylphosphine, TPP; HA, hyaluronic acid; CDDO-Me, Bardoxolone methyl; second mitochondria-derived activator of caspases, SMAC; X-linked inhibitor of apoptosis protein, XIAP; mitochondrial membrane potential (ΔΨ); the mitochondrial permeability transition pore, PTP; DNMT, the DNA, methyltransferase; hair cells, HCs; spiral ganglion neurons, SGNs; PDK1, pyruvate dehydrogenase kinase; mitochondria-distributed Bcl-2, functional conversion peptide NuBCP-9, delivery system, PN9; mPTP, the mitochondrial permeability transition pore; Light chain 3, LC3; Renalcellcarcinoma,RCC.

Dose-dependent production of ROS by derivatives of Betulin and Betulin esters (Ye et al., 2017; Xu et al., 2022) decreased the cell membrane potential and the release of large amounts of cytochrome c following mitochondrial membrane dysfunction initiated the activation of caspase-9, which in turn led to an increase in the number of cleaved segments of the downstream effector caspase-3. As the endpoint of this signaling pathway, cleaved PARP is significantly upregulated and promotes an apoptotic program that effectively inhibits tumor cell proliferation and migration. The chloroform fraction (CMAM) of the ethanolic extract of P. lamblia seeds was effective in inhibiting TNBC (Kariyil et al., 2021) *in vitro* and *in vivo*. This was achieved by apoptosis (Lin et al., 2011) resulting from caspase-activated mitochondria by ROS and by arresting the cell cycle in S phase. The mechanism can be that ROS mediated the activation of the p53 gene and reduced the mitochondrial membrane potential, leading to mitochondrial dysfunction, which in combination led to the apoptosis of NTUB1 cells. Analogously, a triphenyl phosphonium-conjugated glycyrrhetinic acid derivative (Jin et al., 2019), which reduces the mitochondrial membrane potential after ROS generation and activates caspase-3/-9 to induce apoptosis through the mitochondria, significantly inhibits the migration of A549 cells and induces apoptosis in a dose-dependent manner.

Curcumin Derivative B63 (B63) (Zheng et al., 2014) is a strong inhibitor of colon cancer cells. B63 causes elevated ROS due to endoplasmic reticulum stress and mitochondrial dysfunction, followed by upregulation of the expression of the pro-apoptotic proteins Bad and Bim and release of cytochrome c from the mitochondria into the cytoplasm, leading to cleavage of caspase-3 and PARP-1 zymogens. Campesterol has been found to induce the expression of endoplasmic reticulum stress response proteins and promote the generation of ROS in a dose-dependent manner (Bae et al., 2021). Through its modulation of proliferating cell nuclear antigen (PCNA) and PI3K/MAPK signaling pathways, Campesterol has the potential to impede cell growth and disrupt the cell cycle process, ultimately triggering apoptosis. Additionally, it has been observed to augment the anti-cancer effects of cisplatin and paclitaxel while preventing the aggregation of ovarian cancer cells. These effects have been validated in the ES2 and OV90 cell lines. The main compound in pepper, known as cinnamide alkaline-related is pipyine (PIP) (Guo et al., 2021). In HGC-27 cells, PIP induces apoptosis and inhibits cell growth in a dose-dependent manner. This effect is achieved through the modulation of key apoptotic proteins such as Bcl-2, Bax, Cyt-c, caspase-9, and caspase-3, which are influenced by the production of ROS.

CADD522 (Kim et al., 2020), a molecular inhibitor of the runt-related transcription factor 2 (RUNX2) transcription factor, was found to target the enzymatic F1 subunit of the ATP synthase complex to kill cancer cells by regulating ROS levels and thereby inducing apoptosis. The Myoferlin-targeting inhibitor WJ460 was found to induce mitophagy and ROS accumulation (Rademaker et al., 2022), resulting in cell death that was not caused by lipid peroxidation or apoptosis. Additionally, the levels of XC-cystine/glutamate transporter and GPX-4, which are crucial regulators of apoptosis, were significantly reduced in cells treated with WJ460.

To validate its effectiveness, MCF-7 and MDA-MB-231 BC cell lines were utilized. FRI-1 exhibits a pro-survival function and can significantly reduce the maximal oxygen consumption rate (OCR), m, NADH, OGDH, and ATP levels while promoting the production of mitochondrial ROS (Córdova-Delgado et al., 2021).

The mitochondrial targeting product TPP-DOX, which combines doxorubicin (DOX) and triphenylphosphine (TPP), exhibits amphiphilic properties. This poses a challenge for its integration into the nano-captor. To address this issue, the bromide ion of TPP is utilized to link it to hyaluronic acid (HA), leading to the formation of the supramolecular self-assembly structure (HA-Ionic-TPP-DOX). These hydroxyapatite nanocapsules (HA-ion-TPP-DOX) (Liu et al., 2018) have the ability to self-assemble into spherical nanoparticles and are responsive to acidic pH, affecting their morphology and drug release. Compared to free DOX, HA-ion-TPP-DOX displays enhanced localization and accumulation in mitochondria, resulting in elevated levels of ROS and a reduction in mitochondrial membrane potential. The modification produced greater intracellular DOX accumulation and mitochondrial localization compared to free DOX, leading to increased ROS production and slightly reduced mitochondrial membrane potential, increased cytotoxicity and enhanced *in vivo* tumor targeting in MCF-7/ADR cells, and demonstrated better biocompatibility and antitumor effects in tumor-bearing mice and zebrafish.

### 2.3 Targeting antioxidant signaling pathways

#### 2.3.1 Nrf2 endogenous antioxidant pathway

The Nrf2 endogenous antioxidant pathway is a natural system in the body that helps combat free radicals. It is controlled by the Nrf2 trigger, a protein associated with the development of red blood corpuscles and platelets. When there is an increase in the production of reactive oxygen species (ROS), the Nrf2-mediated endogenous antioxidant pathway is activated.

Badosolone and methylbadosolone are semi-synthetic triterpenoids derived from oleanolic acid. These compounds have been found to activate Nrf2 and inhibit the NF-κB pathway. One specific compound, Bardoxolone methyl (CDDO-Me) (Wang Y.-Y. et al., 2014), contains α, β unsaturated carbonyl groups on the A and C rings, allowing it to form reversible adducts with target proteins such as Keap1 and cysteine residues in IκB kinases. Through these interactions, CDDO-Me regulates inflammation, redox homeostasis, cell proliferation, and programmed cell death via the Keap1/Nrf2 and NF-κB signaling pathways.

Furthermore, CDDO-Me has been shown to activate caspase-9, caspase-3, and PARP, leading to a decrease in levels of Bcl-xl and Bcl-2 while increasing levels of Bax and PUMA (Venè et al., 2008). This ultimately results in the release of cytochrome c and induction of apoptosis. Additionally, CDDO-Me can induce autophagy in EC109 and KYse70 cells by inhibiting the PI3K/mTOR pathway (Deeb et al., 2007). It is important to note that the effects of CDDO-Me are dependent on both time and concentration. At low micromolar concentrations, CDDO-Me increases ROS levels and decreases intracellular glutathione levels, leading to apoptosis induction (Wang Y. Y. et al., 2014; Wang et al., 2015). However, further investigation is needed to fully understand the toxicity, adverse effects, and target network of responses associated with CDDO-Me.

Triphenyl phosphonium derivatives of CDDO can induce apoptosis by decreasing or losing mitochondrial membrane potential (Ju et al., 2021a) and is an important marker of mitochondrial apoptosis. The mechanism is that second mitochondria-derived activator of caspases (SMAC) and cytochrome c are released from the mitochondria into the cytoplasm following a decrease in Bcl-2. SMAC binds to X-linked inhibitor of apoptosis protein (XIAP) so that XIAP loses its ability to inhibit caspase family activation, while cytochrome c binds to other molecules to form a caspase activation complex, which induces apoptosis via the caspase-3 and caspase-9 mechanism. The mechanism is that SMAC and cytochrome c are released from the mitochondria into the cytoplasm following a decrease in Bcl-2 (Ju et al., 2021b). SMAC binds to XIAP so that XIAP loses its ability to inhibit caspase family activation (Tian et al., 2021), while cytochrome c binds to other molecules to form a caspase activation complex, which also induces apoptosis via the caspase-3 and caspase-9 mechanism.

#### 2.3.2 Mitochondrial ion pathways

Mitochondrial potassium channels act as regulators of membrane potential and ROS release. In addition to the ATP-dependent potassium channels, there are several other potassium channels localized at different locations within the mitochondria, although their specific physiological and pharmacological properties remain indistinguishable. The presence of different channels regulated by different intracellular factors may provide stimulus-dependent fine-tuning (Szabo et al., 2021) of mitochondrial function, and these channels are all implicated in the regulation of mitochondrial membrane potential (ΔΨ). Since potassium ion influx into the matrix driven by electrochemical gradients leads to depolarization and vice versa, blocking these channels leads to hyperpolarization. Additionally, ΔpH and calcium inward flow may also be regulated by activation or inhibition of K^+^ channels. Moreover, matrix volume is influenced by K^+^ channel function, with both hyperpolarization and depolarization (Malinska et al., 2010) favoring ROS production.

Potassium channel Kv1.3 inhibitors of PAP-1 (Leanza et al., 2017), which is members of a family of psoralenic inhibitors (Vennekamp et al., 2004), block IMM Kv1.3 leading to initial hyperpolarization, release of ROS followed by an increase in ROS levels and secondary depolarization and cell swelling due to the mitochondrial permeability transition pore (PTP) opening, PTP activation (Szabó et al., 2008; Wrzosek et al., 2020) can be produced in mitochondria that lack an endogenous energy source and in the presence of an uncoupler, demonstrating that the development of PTP is not energy reliant. Therefore, ΔΨm dissipation and release of cytochrome c causing apoptosis. AUL12, a Gold (III)-dithiocarbamate complex, is an example that inhibits Complex I of the mitochondrial respiratory chain (Nardon et al., 2015). It induces the production of ROS and activates mitochondrial kinase GSK3-α/β, leading to the opening of the mitochondrial permeability transition pore (MPTP) (Chiara et al., 2012). Amorfrutin C, derived from indigo bush, has been found to reduce the growth of different types of cancer cells, including colon and breast cancer (Weidner et al., 2016). However, this drug has not yet reached the clinical testing stage. Another example of a drug with a similar mechanism is honokiol, extracted from magnolia (Ishitsuka et al., 2005). It can induce cell death in various cancer cells by triggering the opening of MPTP and overcoming resistance mediated by Bcl-2 and Bcl-xL. Honokiol, like Amorfrutin B, activates PPARγ (peroxisome proliferator-activated receptor gamma) (Szabo et al., 2021). Studies have shown that it effectively kills apoptosis-resistant B-cell chronic lymphocytic leukemia (B-CLL) cells and cells from multiple myeloma patients who are resistant to chemotherapy.

The DNA methyltransferase (DNMT) inhibitor RG108 (He et al., 2022) significantly reduces cisplatin-induced damage to hair cells (HCs) and spiral ganglion neurons (SGNs) and protects mitochondrial function by preventing ROS accumulation, resulting in a reduction in apoptosis. By incorporating the triphenyl phosphonium cation fraction dichloroacetophenone derivatives, novel mitochondria-targeted and tumor-specific pyruvate dehydrogenase kinase (PDK1) inhibitors were identified that reduced extracellular acidification and lactate formation (Xu et al., 2018), increased ROS production and depolarized the mitochondrial membrane potential of NCI-H1650 cells, which could act as potential modulators of active mitochondrial OXPHOS and reprogram the glucose metabolic pathway. Recently, it has been discovered that hirsutine, derived from plants, has the ability to fight against cancer in mice with lung cancer (Zhang et al., 2018). It works by activating a series of signals that lead to the dephosphorylation of GSK3β and the opening of MPTP. GSK3β was found to regulate the activity of cyclophilin D, a protein that promotes the opening of pores (Rasola et al., 2010). Importantly, hirsutine did not cause any harm to normal tissues in living organisms.

Therapies that focus on mitochondrial potassium channels might have a crucial role in treating different diseases. To fully explore the potential of mitochondrial potassium channels as drug targets, more precise modulators of these channels are needed.

#### 2.3.3 Bcl-2 and SkQ1

The B-cell lymphoma-2 (Bcl-2) family of proteins plays a crucial role in determining whether a cell will continue to survive or undergo programmed cell death, known as apoptosis (Edlich, 2018). Evasion of apoptosis is one of the key hallmarks of cancer, and elevated levels of pro-survival proteins are a common phenomenon (Sharma et al., 2019) in many types of cancers. Bcl-2 prevents excessive mitochondrial ROS production by reducing mitochondrial, ETC activity. Bcl-2 increases the respiratory rate and cytochrome oxidase activity which leads to the increase of superoxide production.

It has been shown that Bcl-2 has an effect on reducing its interaction with and localization of COX Vα to the mitochondria during oxidative stress. This ultimately results in a decrease in the production of O^2−^ (Chen and Pervaiz, 2007; Low et al., 2010).

Tumor cells could fine-tune the balance between energy requirements and ROS status, and Bcl-2 and COX regulate mitochondrial respiration and the intracellular ROS environment. Higher levels of COX activity, oxygen consumption and mitochondrial respiration were demonstrated in tumor cells overexpressing Bcl-2. Gene silencing and pharmacological inhibition of Bcl-2 confirm these findings. Fascinatingly, Bcl-2 is also able to regulate mitochondrial respiration and COX activity (Chen and Pervaiz, 2007) in the face of increasing levels of ROS triggered by inhibitors of the mitochondrial complex.

The combination of a N-(2 hydroxypropyl) methacrylamide copolymer-based mitochondria-targeted aureomycin delivery system and a mitochondria-distributed Bcl-2 functional conversion peptide NuBCP-9 delivery system (PN9) has been shown to have synergistic effects on tumor regression and metastasis inhibition (Yang et al., 2021). N-Bcl-2 is predominantly found on the outer mitochondrial membrane, as confirmed by fluorescence imaging. Upon the release of PN9, the N9 peptide becomes more active and binds to Bcl-2 on the outer membrane (Luna-Vargas and Chipuk, 2016; O'Neill et al., 2016). This N9 peptide enhances the sensitivity of mitochondria to Mito-Dox, resulting in increased production of reactive oxygen species (ROS) by CII. Subsequently, the mitochondrial permeability transition pore (MPTP) located on the mitochondrial membrane opens, allowing small solutes to enter the mitochondrial matrix, leading to mitochondrial dysfunction and cell apoptosis. The co-administration of these systems disrupts intra-mitochondrial homeostasis in multiple ways, offering a promising approach for the treatment of primary and metastatic breast cancer.

Azathioprine concentrations-dependently inhibits mitochondrial metabolic activity to a degree that causes mitochondrial membrane damage, contributes to increased oxidative stress on the rough endoplasmic reticulum by inducing increased ROS levels, and induces apoptosis (Trybus et al., 2022). Azathioprine increases DNA breakage and induces apoptosis by inactivating the anti-apoptotic protein Bcl-2 in HeLa cells, inducing the production of multinucleon and vacuoles, which direct the apoptotic pathway to degrade the endoplasmic reticulum. Induction of autophagy by increasing Light chain 3 (LC3) protein levels according to the principles of the assay used. LC3 is a cytoplasmic protein involved in autophagosome formation during autophagy, which is transferred from the cytoplasm to the interior of the autophagosome.

SkQ1 is currently the world’s only cellular mitochondria-targeted antioxidant that protects cells throughout the body, reaching directly into cellular mitochondria to scavenge excess free radicals, effectively reducing free radical attack on mitochondria and cells while enhancing the efficiency of mitochondria in burning energy (Bazhin et al., 2016). SkQ1 upregulates the production of anxiogenic factors based on the fact that ROS regulate tumor angiogenesis and all protocols treated with SkQ1 reduce the percentage of NKT cells, which are a major cause of autoimmune disease and cause disorders of the immune system. SkQ1 has shown promising results in various disease models, including stroke, autoimmune arthritis, and neurobehavioral disorders (Zielonka et al., 2017).

## 3 Conclusion

Mitochondria often exhibit metabolic abnormalities in tumor cells, converting the normal metabolic pattern to glycolysis under aerobic conditions and up- or down-regulating OXPHOS levels, resulting in adverse consequences such as increased ROS levels, massive production of free radicals, dysregulation of ion channels and other metabolic shifts leading to genomic instability, altered signaling pathways and changes in downstream proteins and enzymes, leading to a “vicious circle” (Yang et al., 2016b) between mitochondria, ROS, genomic instability and cancer development. Based on the multifaceted functions of mitochondria in energy metabolism, apoptosis regulation and cell signaling, reducing the toxic effects on normally metabolizing cells is an area where mitochondria-targeted agents have significant therapeutic potential compared to other targeted anti-cancer agents. However, most of the mitochondria-targeting agents are at the experimental stage and no reliable clinical data are available. In addition, there are few studies on whether some drugs such as atovaquone and azathioprine, which have their own pharmacological effects, can be combined. Hence, in order to fully exploit the therapeutic potential of mitochondrial targeted agents, it is essential to fully understand the mechanisms of action of mitochondrial-targeting agents. This study provides guidance to support the development of novel anti-cancer targeted agents.
