# Supplementary material for: The mechanisms of action of mitochondrial targeting agents in cancer: inhibiting oxidative phosphorylation and inducing apoptosis
Source: Front Pharmacol. 2023 Oct 25;14:1243613. doi: 10.3389/fphar.2023.1243613 (PMC10635426; doi:10.3389/fphar.2023.1243613)
Supplement: Supplementary file 1 [file DataSheet2.PDF]

Supplementary Material

1 SUPPLEMENTARY TABLES AND FIGURES

Abbreviations: ROS, reactive oxygen species; OXPHOS, oxidative phosphorylation; TME, tumor microenvironment; ETC, Electron transport chain; Complex I, CI; Complex II, CII; Complex III, CIII; Complex IV, CIV; Complex V, CV; Succinate dehydrogenase, SDH; succinate dehydrogenase complex subunit A, SDHA ;succinate dehydrogenase complex subunit B, SDHB ;succinate dehydrogenase complex subunit C, SDHC ; succinate coenzyme q oxidoreductase, SQR; coenzyme q ,CoQ; Factor-related Apoptosis ligand, FasL; tumor necrosis factor, TNF; acidification in the cytoplasm, pH-c; acidification in the mitochondria , pH-m; triphosphopyridine nucleotide ,NADPH ;glutathione, GSH; Glutathione-oxidized, GSSG; oxoglutarate dehydrogenase ,OGDH;TCA, tricarboxylic acid cycle; adenosine triphosphate, ATP; adenosine diphosphate ,ADP; phosphoric acid ,Pi; nicotinamide adenine dinucleotide ,NADH; The amino acids arginine 21, R21; lysine 108 ,K108; mitochondrial ribosomal protein S23 ,MRPS23; the enzymes protein arginine methyltransferase 7 ,PRMT7; set domain-containing protein 6 ,SETD6; Mito-Met, metformin; DFO, desferrioxamine; TNBC, triple-negative breast cancer; B87, BAY87-2243; DMKG, dimethyl α-ketoglutarate; isocitrate dehydrogenase 1 ,IDH1; mousedouble minute 2, MDM2; 2-Deoxy-Dglucose, 2-DG;TP-53, tumor suppressor protein p53; poly (ADP-ribose) polymerase 1, PARP1; apoptosisinducing factor 1, AIFM1; apyrimidinic endodeoxyribonuclease 1, APEX1; OPi, IAC-S010759; AML, acute myeloid leukemia; NMP, nucleotide monophosphate; NTP, nucleotide triphosphate; hypoxia-inducible factor, HIF; adenosine 5‘-monophosphate-activated protein kinase ,AMPK; Liver kinase B1,LKB1; NSCLC, non-small cell lung cancer; ERK, extracellular regulated protein kinases; Mito- MGN, Mito-magnolol; OCR, oxygen consumption rate; protein kinase B, AKT; The novel L. origanoides extract LOE; Mito-Hu, mitochondria-targeted hydroxyurea; Mito-LND, Mito-lonidamine; Mito-Tam, Targeting tamoxifen; SMAD family member 4 Gene, SMAD4; Transforming Growth Factor-β, TGF-β; alpha-vitamin E succinate,α-TOS; UbQ, ubiquinone; γ-T3, γ-Tocotrienol; Mitochondrially targeted vitamin E succinate ,Mito-VES; B cell lymphoma 2 (BCL2) homology domain 3 , BH3; dihydroorotate dehydrogenase ,DHODH; ginsenoside Rh2,G-Rh2; high grade gliomas, HGGs; Mitochondria-targeted atovaquone ,Mito-ATO; Programmed Death 1, PD-1;mitochondria-targeted carboxy-proxyl,Mito-CP; MTC, medullary thyroid carcinoma; cytochrome c oxidase IV , COX IV; myeloid cell leukemia-1, Mcl-1; a synthetic cationic acetamide analog ,Mito-CP-Ac; Mitoquinone ,Mito-Q; MMP, mitochondrial membrane potential; Uncoupling protein 2, UCP2; solute carrier family 25, SLC25; mitochondria-targeted drugs ,MTD; NOxs, NADPH oxidases; alpha-ketoglutarate MB-453 (erbB2-high) and MCF7DD9 cells with transcriptionally inactive p53; human colorectal cells HCT116; human neuroblastoma TetN21 cells; human non-small-cell lung carcinoma cells H1299; human cervical cancer cells HeLa; mouse mesothelioma cells AE17; human

nonmalignant mesothelial cells Met-5A; human fibroblasts A014578; rat ventricular myocyte-like cells HL1; and mouse atrial myocyte-like cells H9c2. bearing xenotransplantation drug resistant human chronic myeloid leukemia K562/ADR tumor, and the tumor inhibition rate was up to 82.38% Mito-CP-Ac CIII and inhibit mitochondrial oxygen consumption MiaPaCa-2, PANC-1, MCF-7, MDAMB-231, MCF-10A and A431 Cells Combined use of 2-DG would synergistically enhance cytotoxic selectivity in cancer cells (Zhou et al., 2022) Mito-Q Induction of mitochondrial uncoupling MCF-7, MCF-10A, and MDA-MB231 cells Combined use of 2-DG would synergistically enhance cytotoxic selectivity in cancer cells (Cheng et al., 2012) Frontiers 25 10 dehydrogenase complex, A-KGDH; 2-oxoglutarate dehydrogenase complex ,OGDC; pyruvate dehydrogenase , PDH; COX, cytochrome c oxidase; CMAM, the chloroform fraction; B63, Curcumin Derivative B63; proliferating cell nuclear antigen, PCNA; pipyine ,PIP; runt-related transcription factor 2,RUNX2; doxorubicin, DOX; triphenylphosphine, TPP; HA, hyaluronic acid; CDDO-Me, Bardoxolone methyl; second mitochondria-derived activator of caspases, SMAC; X-linked inhibitor of apoptosis protein, XIAP; mitochondrial membrane potential (ΔΨ)； the mitochondrial permeability transition pore, PTP; DNMT, the DNA methyltransferase; hair cells, HCs; spiral ganglion neurons, SGNs; PDK1, pyruvate dehydrogenase kinase; mitochondria-distributed Bcl-2 functional conversion peptide NuBCP-9 delivery system, PN9; mPTP, the mitochondrial permeability transition pore; Light chain 3, LC3.
