# Supplementary material for: The mechanisms of action of mitochondrial targeting agents in cancer: inhibiting oxidative phosphorylation and inducing apoptosis
Source: Front Pharmacol. 2023 Oct 25;14:1243613. doi: 10.3389/fphar.2023.1243613 (PMC10635426; doi:10.3389/fphar.2023.1243613)
Supplement: Supplementary file 2 [file Table1.DOCX]

**Table 1. Sites of action of agents targeting mitochondrial complexes and conclusions**

| Drugs | Effect target | Application | Reference |
| --- | --- | --- | --- |
| Mitomycin metformin | CI | PANC-1、TNBC | (Cheng et al., 2019b) |
| Mito-DFO | CI | MCF-7、MDA-MB-231 | (Sandoval-Acuna et al., 2021) |
| BAY87-2243 | CI | - | (Sica et al., 2019) |
| IAC-S010759 (OPi) | CI | Multiple MAPKi-resistant BRAF-mutant melanoma models, acute myeloid leukemia (AML) models | (Molina et al., 2018; Vashisht Gopal et al., 2019) |
| EVT-701 | CI | In vitro and in vivo efficacy of OXPHOS-Eµ-Myc lymphoma in mouse, NSCLC, and NH-B-cell lymphoma models | (Luna Yolba et al., 2021) |
| ME-143/ME-344 | CI and mildly inhibit CIII | HEK293T human embryonic kidney | (Lim et al., 2015) |
| Mito-MGN | CI | B16-F10、B16-F0 | (Cheng et al., 2020; AbuEid et al., 2021) |
| Lippia organoids extract | CI | MDA-MB-231 | (Raman et al., 2017; Raman et al., 2018) |
| Mitochondria-targeted hydroxyurea（Mito-Hu） | CI | Miapaca2 | (Boddapati et al., 2008; Cheng et al., 2021) |
| Mito-lonidamine（Mito-LND） | CI | A549、H2030BrM3 | (Cheng et al., 2019a) |
| Mito-Tamoxifen | Inhibit CI and CII | HCT116、DU 145、MCF-7 | (Rohlenova et al., 2017; Ezrova et al., 2021) |
| α-TOS | CII | MCF-7、MDA-MB-453、NSC、B9rec、B10、B1 | (Dong et al., 2008) |
| γ-Tocotrienol（γ-T3） | CII | SGC-7901、MGC-803 | (Wang et al., 2019) |
| Mito-VES | CII | human T lymphoma Jurkat, Bax-Jurkat, and Bax-/Bak-Jurkat cells; human mesothelioma  cells Meso2, Ist-Mes-1, Ist-Mes-2, and MM-BI; human breast cancer  cells MCF7 (erbB2-low) and MDA-MB-453 (erbB2-high) and MCF7DD9 cells with transcriptionally inactive p53; human colorectal cells HCT116; human neuroblastoma TetN21 cells; human non-small-cell lung carcinoma cells H1299; human cervical cancer cells HeLa; mouse mesothelioma cells AE17; human nonmalignant mesothelial cells Met-5A; human fibroblasts A014578; rat ventricular myocyte-like cells HL1; and mouse atrial myocyte-like cells H9c2. | (Dong et al., 2011; Liang et al., 2021) |
| GinsenosideRh2 | CI, III and V | Hela、C33A、End1/e6e7 Cells | (Liu et al., 2021) |
| Mito-Atovaquone | CI, CIII | LKR13、unscc680、LKR13-luc | (Mudassar et al., 2020; Huang et al., 2022) |
| Capsaicin | CI, CIII | BxPC-3、AsPC-1 | (Pramanik et al., 2011) |
| Mitochondria-targeted carboxy-proxyl (Mito-CP) | COX IV、Mcl-1 were dramatically down-regulated | TT、MZ-CRC-1 | (Starenki and Park, 2013; Cheng et al., 2015; Hong et al., 2017) |
| Mito-CP-Ac | CIII | MiaPaCa-2、 PANC-1、 MCF-7、 MDA-MB-231、 MCF-10A and A431 Cells | (Zhou et al., 2022) |

Table 2. Other mechanisms of mitochondrial targeting agents and applications

| Drugs | Mechanism | Application | Reference |
| --- | --- | --- | --- |
| Mito-Q | Induction of mitochondrial uncoupling | MCF-7, MCF-10A, and MDA-MB-231 cells | (Cheng et al., 2012) |
| Lupane Triterpenoid Derivatives | The dose-dependent induction of ROS production reduced the cell membrane potential | K562、A549、ECA-109、HepG2、HL-7702、HL-60 | (Xu et al., 2022) |
| 3-O-(3'-acetylphenylacetate)-betulin with triphenyl phosphonium | Arrest of the tumor cell at the G2/M phase, caused ROS overproduction, decreased ψM, and induced apoptosis via the  mitochondria pathway. | A549、U87、Hela、MDA-MB231、HCT116 | (Kariyil et al., 2021) |
| Chloroform Fraction of Methanolic Extract of Seeds of Annona muricata (CMAM) | To induct S Phase arrest and ROS dependent caspase activated mitochondria-mediated  apoptosis | MDA-MB-231 | (Lin et al., 2011) |
| 18b-Glycyrrhetinic acid derivatives | The dose-dependent induction of cell apoptosis | A549、U87、Hela、MDA-MB231、HCT116、NCM460 | (Jin et al., 2019) |
| Triphenyl phosphonium conjugated glycyrrhetinic  acid derivatives | Apoptosis cells through the mitochondrial  pathway via the collapse of mitochondrial membrane potential, reactive oxygen species production and the  activation of caspase-9 and caspase-3 | HepG-2、A549、MCF-7、HT-29、A2780、HL-7702 | (Zheng et al., 2014) |
| Curcumin Derivative B63（B63） | ROS elevation caused by ER stress and mitochondrial dysfunction | SW620、SW480、HCT116、HIEC | (Kim et al., 2020) |
| CADD522 | Regulation of ROS levels induces apoptosis | Hs578t、RUNX2 KD、MCF7-RUNX2 | (Liu et al., 2018) |
| HA-ionic-TPP-DOX | Increased ROS production and slightly decreased mitochondrial membrane potential | MCF-7/ADR | (Wang et al., 2014) |
| Bardoxolone methyl (CDDO-Me) | Apoptosis was induced by increasing ROS and decreasing intracellular glutathione levels | EC109、KYse70 | (Wang et al., 2015; Ju et al., 2021) |
| Triphenyl phosphonium derivatives of CDDO | Mitochondrial membrane potential decreased and cell apoptosis was induced | MCF-7 | (Szabo et al., 2021) |
| Mito-chondriotropic PAP-1 Derivatives | Blockade of IMM Kv1.3 resulted in an initial hyperpolarization and apoptosis caused by cytochrome C release after ROS release | Jurkat T Cells、Primary pathology CD19+/ CD5+ B Cells, B16F10 Melanoma Cells, Pathological B-CLL Cells, | (He et al., 2022) |
| DNA methyltransferase (DNMT) inhibitor RG108 | RG108 treatment could reduce ROS accumulation and inhibit apoptosis, which is mediated, at least partially, through LRP1ePI3K/AKT signaling pathway | HEI-OC1 | (Xu et al., 2018) |
| Pyruvate Dehydrogenase Kinase (PDK1) Inhibitors | The extracellular acidification rate and lactate formation were decreased, and ROS production was increased | NCI-H1650 | (Sharma et al., 2019) |
| Mitochondrial targeted Doxorubicin (Dox) delivery System based on N-(2-hydroxypropyl) methyl acrylamide copolymer and Mitochondrial Distributed Bcl-2 Function switching peptide NuBCP-9 Delivery System (PN9) | Imbalance of mitochondrial homeostasis | 4T1 | (O'Neill et al., 2016) |
| Azelastine | Inducing ROS levels to increase is helpful to increase oxidative stress and stress in rough endoplasmic reticulum and induce apoptosis | Hela | (Bazhin et al., 2016) |
| SkQ1 | Scavenging excess free radicals in mitochondria | Mia-Paca、Dan-G | (Yang et al., 2016) |

**Table 3. Research progress and effect of mitochondrial targeting agents in combination with other drugs**

| Drugs | Effect target | Application | Drug combination | Reference |
| --- | --- | --- | --- | --- |
| Mitomycin metformin | CI | PANC-1、TNBC | Iron chelators, DFX, and deferoxamine synergistically inhibited proliferation | (Cheng et al., 2019b) |
| BAY87-2243 | CI | - | Combined with DMKG affects metabolic activity | (Sica et al., 2019) |
| Mito-Tamoxifen | CI, CII | HCT116、DU 145、MCF-7 | Doxorubicin-induced apoptosis was attenuated | (Rohlenova et al., 2017; Ezrova et al., 2021) |
| Mito-VES | CII | human T lymphoma Jurkat, Bax-Jurkat, and Bax-/Bak-Jurkat cells; human mesothelioma  cells Meso2, Ist-Mes-1, Ist-Mes-2, and MM-BI; human breast cancer  cells MCF7 (erbB2-low) and MDA-MB-453 (erbB2-high) and MCF7DD9 cells with transcriptionally inactive p53; human colorectal cells HCT116; human neuroblastoma TetN21 cells; human non-small-cell lung carcinoma cells H1299; human cervical cancer cells HeLa; mouse mesothelioma cells AE17; human nonmalignant mesothelial cells Met-5A; human fibroblasts A014578; rat ventricular myocyte-like cells HL1; and mouse atrial myocyte-like cells H9c2. | The combination of Mito-VES and doxorubicin hydrochloride significantly enhanced the anti-tumor effect of dual-loaded nanocapsules in nude mice bearing xenotransplantation drug resistant human chronic myeloid leukemia K562/ADR tumor, and the tumor inhibition rate was up to 82.38% | (Dong et al., 2011; Liang et al., 2021) |
| Mito-CP-Ac | CIII and  inhibit mitochondrial oxygen consumption | MiaPaCa-2, PANC-1, MCF-7, MDA-MB-231, MCF-10A and A431 Cells | Combined use of 2-DG would synergistically enhance cytotoxic selectivity in cancer cells | (Zhou et al., 2022) |
| Mito-Q | Induction of mitochondrial uncoupling | MCF-7, MCF-10A, and MDA-MB-231 cells | Combined use of 2-DG would synergistically enhance cytotoxic selectivity in cancer cells | (Cheng et al., 2012) |

**Reference**

AbuEid, M., McAllister, D.M., McOlash, L., Harwig, M.C., Cheng, G., Drouillard, D., et al. (2021). Synchronous effects of targeted mitochondrial complex I inhibitors on tumor and immune cells abrogate melanoma progression. *iScience* 24(6)**,** 102653.

Bazhin, A.V., Yang, Y., D'Haese, J.G., Werner, J., Philippov, P.P., and Karakhanova, S. (2016). The novel mitochondria‐targeted antioxidant SkQ1 modulates angiogenesis and inflammatory micromilieu in a murine orthotopic model of pancreatic cancer. *International Journal of Cancer* 139(1)**,** 130-139.

Boddapati, S.V., D’Souza, G.G., Erdogan, S., Torchilin, V.P., and Weissig, V. (2008). Organelle-targeted nanocarriers: specific delivery of liposomal ceramide to mitochondria enhances its cytotoxicity in vitro and in vivo. *Nano letters* 8(8)**,** 2559-2563.

Cheng, G., Hardy, M., Topchyan, P., Zander, R., Volberding, P., Cui, W., et al. (2021). Mitochondria-targeted hydroxyurea inhibits OXPHOS and induces antiproliferative and immunomodulatory effects. *iScience* 24(6)**,** 102673.

Cheng, G., Hardy, M., Zielonka, J., Weh, K., Zielonka, M., Boyle, K.A., et al. (2020). Mitochondria-targeted magnolol inhibits OXPHOS, proliferation, and tumor growth via modulation of energetics and autophagy in melanoma cells. *Cancer treatment and research communications* 25**,** 100210.

Cheng, G., Zhang, Q., Pan, J., Lee, Y., Ouari, O., Hardy, M., et al. (2019a). Targeting lonidamine to mitochondria mitigates lung tumorigenesis and brain metastasis. *Nature communications* 10(1)**,** 2205.

Cheng, G., Zielonka, J., Dranka, B.P., McAllister, D., Mackinnon Jr, A.C., Joseph, J., et al. (2012). Mitochondria-targeted drugs synergize with 2-deoxyglucose to trigger breast cancer cell death. *Cancer research* 72(10)**,** 2634-2644.

Cheng, G., Zielonka, J., Hardy, M., Ouari, O., Chitambar, C.R., Dwinell, M.B., et al. (2019b). Synergistic inhibition of tumor cell proliferation by metformin and mito-metformin in the presence of iron chelators. *Oncotarget* 10(37)**,** 3518.

Cheng, G., Zielonka, J., McAllister, D., Hardy, M., Ouari, O., Joseph, J., et al. (2015). Antiproliferative effects of mitochondria-targeted cationic antioxidants and analogs: Role of mitochondrial bioenergetics and energy-sensing mechanism. *Cancer letters* 365(1)**,** 96-106.

Dong, L.-F., Jameson, V.J., Tilly, D., Prochazka, L., Rohlena, J., Valis, K., et al. (2011). Mitochondrial targeting of α-tocopheryl succinate enhances its pro-apoptotic efficacy: a new paradigm for effective cancer therapy. *Free Radical Biology and Medicine* 50(11)**,** 1546-1555.

Dong, L.-F., Low, P., Dyason, J.C., Wang, X.-F., Prochazka, L., Witting, P.K., et al. (2008). α-Tocopheryl succinate induces apoptosis by targeting ubiquinone-binding sites in mitochondrial respiratory complex II. *Oncogene* 27(31)**,** 4324-4335.

Ezrova, Z., Nahacka, Z., Stursa, J., Werner, L., Vlcak, E., Kralova Viziova, P., et al. (2021). SMAD4 loss limits the vulnerability of pancreatic cancer cells to complex I inhibition via promotion of mitophagy. *Oncogene* 40(14)**,** 2539-2552.

He, Y., Zheng, Z., Liu, C., Li, W., Zhao, L., Nie, G., et al. (2022). Inhibiting DNA methylation alleviates cisplatin-induced hearing loss by decreasing oxidative stress-induced mitochondria-dependent apoptosis via the LRP1–PI3K/AKT pathway. *Acta Pharmaceutica Sinica B* 12(3)**,** 1305-1321.

Hong, S.-K., Starenki, D., Wu, P.-K., and Park, J.-I. (2017). Suppression of B-RafV600E melanoma cell survival by targeting mitochondria using triphenyl-phosphonium-conjugated nitroxide or ubiquinone. *Cancer biology & therapy* 18(2)**,** 106-114.

Huang, M., Xiong, D., Pan, J., Zhang, Q., Wang, Y., Myers, C.R., et al. (2022). Prevention of Tumor Growth and Dissemination by In Situ Vaccination with Mitochondria‐Targeted Atovaquone. *Advanced Science* 9(12)**,** 2101267.

Jin, L., Dai, L., Ji, M., and Wang, H. (2019). Mitochondria-targeted triphenylphosphonium conjugated glycyrrhetinic acid derivatives as potent anticancer drugs. *Bioorganic chemistry* 85**,** 179-190.

Ju, W., Li, N., Wang, J., Yu, N., Lei, Z., Zhang, L., et al. (2021). Design and synthesis of novel mitochondria-targeted CDDO derivatives as potential anti-cancer agents. *Bioorganic Chemistry* 115**,** 105249.

Kariyil, B.J., Ayyappan, U., Gopalakrishnan, A., and George, A.J. (2021). Chloroform fraction of methanolic extract of seeds of Annona muricata induce S phase arrest and ROS dependent caspase activated mitochondria-mediated apoptosis in triple-negative breast cancer. *Anti-Cancer Agents in Medicinal Chemistry (Formerly Current Medicinal Chemistry-Anti-Cancer Agents)* 21(10)**,** 1250-1265.

Kim, M.S., Gernapudi, R., Cedeño, Y.C., Polster, B.M., Martinez, R., Shapiro, P., et al. (2020). Targeting breast cancer metabolism with a novel inhibitor of mitochondrial ATP synthesis. *Oncotarget* 11(43)**,** 3863.

Liang, L., Peng, Y., and Qiu, L. (2021). Mitochondria-targeted vitamin E succinate delivery for reversal of multidrug resistance. *Journal of Controlled Release* 337**,** 117-131.

Lim, S.C., Carey, K.T., and McKenzie, M. (2015). Anti-cancer analogues ME-143 and ME-344 exert toxicity by directly inhibiting mitochondrial NADH: ubiquinone oxidoreductase (Complex I). *American journal of cancer research* 5(2)**,** 689.

Lin, K.-W., Huang, A.-M., Hour, T.-C., Yang, S.-C., Pu, Y.-S., and Lin, C.-N. (2011). 18β-Glycyrrhetinic acid derivatives induced mitochondrial-mediated apoptosis through reactive oxygen species-mediated p53 activation in NTUB1 cells. *Bioorganic & medicinal chemistry* 19(14)**,** 4274-4285.

Liu, H.-n., Guo, N.-n., Guo, W.-w., Huang-Fu, M.-y., Vakili, M.R., Chen, J.-j., et al. (2018). Delivery of mitochondriotropic doxorubicin derivatives using self-assembling hyaluronic acid nanocarriers in doxorubicin-resistant breast cancer. *Acta Pharmacologica Sinica* 39(10)**,** 1681-1692.

Liu, Y., Yu, S., Xing, X., Qiao, J., Yin, Y., Wang, J., et al. (2021). Ginsenoside Rh2 stimulates the production of mitochondrial reactive oxygen species and induces apoptosis of cervical cancer cells by inhibiting mitochondrial electron transfer chain complex. *Molecular Medicine Reports* 24(6)**,** 1-14.

Luna Yolba, R., Visentin, V., Hervé, C., Chiche, J., Ricci, J.E., Méneyrol, J., et al. (2021). EVT‐701 is a novel selective and safe mitochondrial complex 1 inhibitor with potent anti‐tumor activity in models of solid cancers. *Pharmacology Research & Perspectives* 9(5)**,** e00854.

Molina, J.R., Sun, Y., Protopopova, M., Gera, S., Bandi, M., Bristow, C., et al. (2018). An inhibitor of oxidative phosphorylation exploits cancer vulnerability. *Nature medicine* 24(7)**,** 1036-1046.

Mudassar, F., Shen, H., O’Neill, G., and Hau, E. (2020). Targeting tumor hypoxia and mitochondrial metabolism with anti-parasitic drugs to improve radiation response in high-grade gliomas. *Journal of Experimental & Clinical Cancer Research* 39(1)**,** 1-17.

O'Neill, K.L., Huang, K., Zhang, J., Chen, Y., and Luo, X. (2016). Inactivation of prosurvival Bcl-2 proteins activates Bax/Bak through the outer mitochondrial membrane. *Genes & development* 30(8)**,** 973-988.

Pramanik, K.C., Boreddy, S.R., and Srivastava, S.K. (2011). Role of mitochondrial electron transport chain complexes in capsaicin mediated oxidative stress leading to apoptosis in pancreatic cancer cells. *PloS one* 6(5)**,** e20151.

Raman, V., Aryal, U.K., Hedrick, V., Ferreira, R.M., Fuentes Lorenzo, J.L., Stashenko, E.E., et al. (2018). Proteomic analysis reveals that an extract of the plant Lippia origanoides suppresses mitochondrial metabolism in triple-negative breast cancer cells. *Journal of Proteome Research* 17(10)**,** 3370-3383.

Raman, V., Fuentes Lorenzo, J.L., Stashenko, E.E., Levy, M., Levy, M.M., and Camarillo, I.G. (2017). Lippia origanoides extract induces cell cycle arrest and apoptosis and suppresses NF-κB signaling in triple-negative breast cancer cells. *International Journal of Oncology* 51(6)**,** 1801-1808.

Rohlenova, K., Sachaphibulkij, K., Stursa, J., Bezawork-Geleta, A., Blecha, J., Endaya, B., et al. (2017). Selective disruption of respiratory supercomplexes as a new strategy to suppress Her2high breast cancer. *Antioxidants & redox signaling* 26(2)**,** 84-103.

Sandoval-Acuna, C., Torrealba, N., Tomkova, V., Jadhav, S.B., Blazkova, K., Merta, L., et al. (2021). Targeting mitochondrial iron metabolism suppresses tumor growth and metastasis by inducing mitochondrial dysfunction and mitophagy. *Cancer Research* 81(9)**,** 2289-2303.

Sharma, A., Boise, L.H., and Shanmugam, M. (2019). Cancer metabolism and the evasion of apoptotic cell death. *Cancers* 11(8)**,** 1144.

Sica, V., Bravo-San Pedro, J.M., Izzo, V., Pol, J., Pierredon, S., Enot, D., et al. (2019). Lethal poisoning of cancer cells by respiratory chain inhibition plus dimethyl α-ketoglutarate. *Cell reports* 27(3)**,** 820-834. e829.

Starenki, D., and Park, J.-I. (2013). Mitochondria-targeted nitroxide, Mito-CP, suppresses medullary thyroid carcinoma cell survival in vitro and in vivo. *The Journal of Clinical Endocrinology & Metabolism* 98(4)**,** 1529-1540.

Szabo, I., Zoratti, M., and Biasutto, L. (2021). Targeting mitochondrial ion channels for cancer therapy. *Redox Biology* 42**,** 101846.

Vashisht Gopal, Y., Gammon, S., Prasad, R., Knighton, B., Pisaneschi, F., Roszik, J., et al. (2019). A Novel Mitochondrial Inhibitor Blocks MAPK Pathway and Overcomes MAPK Inhibitor Resistance in MelanomaOxPhos Inhibition Counteracts MAPK Inhibitor Resistance. *Clinical Cancer Research* 25(21)**,** 6429-6442.

Wang, H., Luo, J., Tian, W., Yan, W., Ge, S., Zhang, Y., et al. (2019). γ-Tocotrienol inhibits oxidative phosphorylation and triggers apoptosis by inhibiting mitochondrial complex I subunit NDUFB8 and complex II subunit SDHB. *Toxicology* 417**,** 42-53.

Wang, Y.-Y., Yang, Y.-X., Zhao, R., Pan, S.-T., Zhe, H., He, Z.-X., et al. (2015). Bardoxolone methyl induces apoptosis and autophagy and inhibits epithelial-to-mesenchymal transition and stemness in esophageal squamous cancer cells. *Drug design, development and therapy* 9**,** 993.

Wang, Y.-Y., Yang, Y.-X., Zhe, H., He, Z.-X., and Zhou, S.-F. (2014). Bardoxolone methyl (CDDO-Me) as a therapeutic agent: an update on its pharmacokinetic and pharmacodynamic properties. *Drug design, development and therapy***,** 2075-2088.

Xu, B., Yu, Z., Xiang, S., Li, Y., Zhang, S.-L., and He, Y. (2018). Rational design of mitochondria-targeted pyruvate dehydrogenase kinase 1 inhibitors with improved selectivity and antiproliferative activity. *European Journal of Medicinal Chemistry* 155**,** 275-284.

Xu, G., Xu, X., Liu, J., Jia, Q., Ke, C., Zhang, H., et al. (2022). Mitochondria‐Targeted Triphenylphosphonium Conjugated C‐3 Modified Betulin: Synthesis, Antitumor Properties and Mechanism of Action. *ChemMedChem* 17(4)**,** e202100659.

Yang, Y., Karakhanova, S., Hartwig, W., D'Haese, J.G., Philippov, P.P., Werner, J., et al. (2016). Mitochondria and mitochondrial ROS in cancer: novel targets for anticancer therapy. *Journal of cellular physiology* 231(12)**,** 2570-2581.

Zheng, A., Li, H., Wang, X., Feng, Z., Xu, J., Cao, K., et al. (2014). Anticancer effect of a curcumin derivative B63: ROS production and mitochondrial dysfunction. *Current Cancer Drug Targets* 14(2)**,** 156-166.

Zhou, D., Zhuan, Q., Luo, Y., Liu, H., Meng, L., Du, X., et al. (2022). Mito-Q promotes porcine oocytes maturation by maintaining mitochondrial thermogenesis via UCP2 downregulation. *Theriogenology* 187**,** 205-214.
