# Supplementary material for: The mechanisms of action of mitochondrial targeting agents in cancer: inhibiting oxidative phosphorylation and inducing apoptosis
Source: Front Pharmacol. 2023 Oct 25;14:1243613. doi: 10.3389/fphar.2023.1243613 (PMC10635426; doi:10.3389/fphar.2023.1243613)
Supplement: Supplementary file 3 [file DataSheet1.PDF]

Supplementary Material

1 SUPPLEMENTARY TABLES AND FIGURES

Table S1. The structures of the discussed mitochondria-targeted compounds

Chemical composition	Effect target	Structural formula	Reference
Metformin	CI	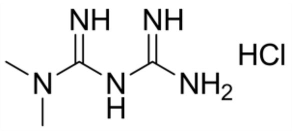	(Bridges et al., 2014)
Mito-metformin	CI	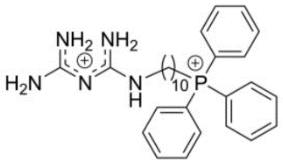	(Cheng et al., 2019b)
Mito- desferrioxamine	CI	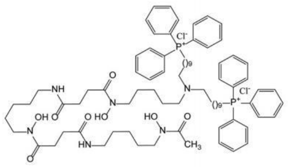	(Sandoval-Acuna et al., 2021)
BAY-872243	CI	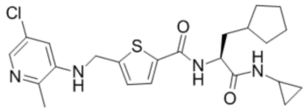	(Sica et al., 2019)
IACS-010759 (OPi)	CI	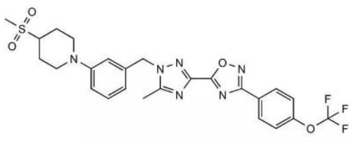	(Molina et al., 2018)
EVT-701	CI	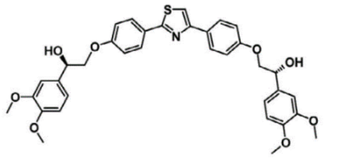	(Luna Yolba et al., 2021)
ME-143/ME-344	CI and mildly inhibit CIII	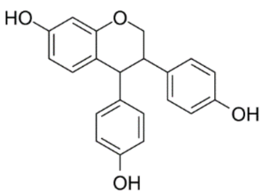	(Lim et al., 2015)

Mito-MGN	CI	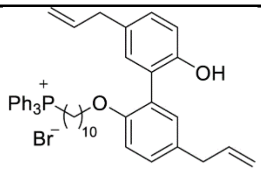	(Cheng et al., 2020)
Mitochondria-targeted hydroxyurea （Mito-Hu）	CI	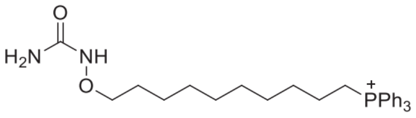	(Cheng et al., 2021)
Mito-lonidamine（Mito-LND）	CI	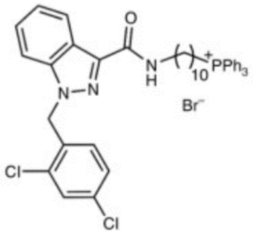	(Cheng et al., 2019a)
α-TOS	CII	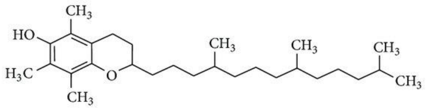	(Dong et al., 2008)
γ-Tocotrienol (γ-T3)	CII	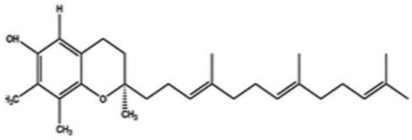	(Wang et al., 2019)
Mito-VES	CII	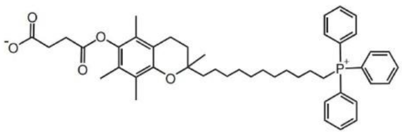	(Dong et al., 2011; Liang et al., 2021)
GinsenosideRh2	CI, III and V	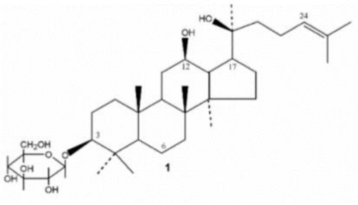	(Liu et al., 2021)
Capsaicin	CI, CIII	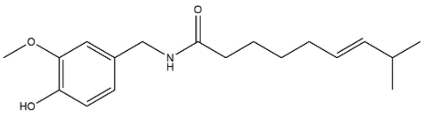	(Pramanik et al., 2011)
Mitochondria-targeted carboxy-proxyl (Mito-CP)	COX IV 、 Mcl-1 were dramatically downregulated	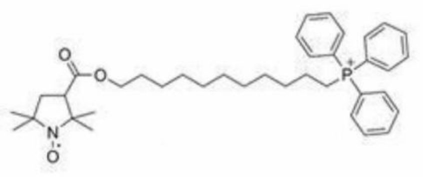	(Starenki and Park, 2013; Cheng et al., 2015; Hong et al., 2017)
Mito-CP-Ac	CIII	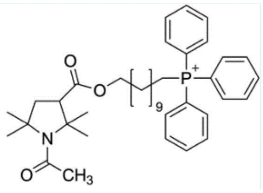	(Zhou et al., 2022)

frontiers

Betulin and Betulin esters	Apoptosis and ROS	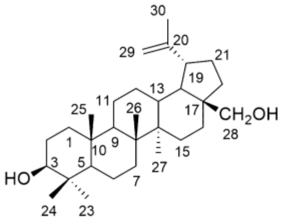	(Ye et al., 2017)
Triphenyl phosphonium-conjugated glycyrrhetinic acid derivative	Apoptosis and ROS	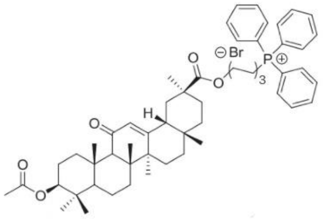	(Jin et al., 2019)
Curcumin Derivative B63 (B63)	Apoptosis and ROS	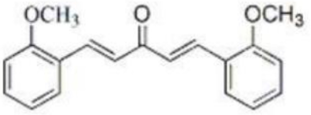	(Zheng et al., 2014)
An analogue of the natural para-kaurane diterpene (DS2)	Apoptosis and ROS	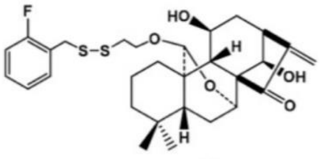	(Ma et al., 2016)
CADD522	Apoptosis and ROS	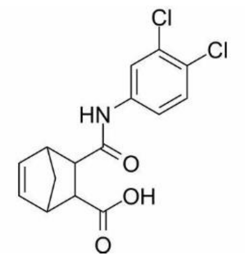	(Kim et al., 2020)
WJ460	Apoptosis and ROS	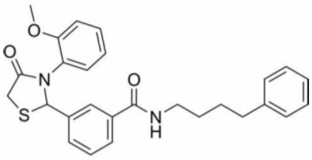	(Rademaker et al., 2022)
FRI-1	Apoptosis and ROS	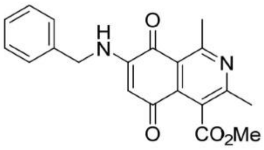	(Córdova-Delgado et al., 2021)
HA-ionic-TPP-DOX	Apoptosis and ROS	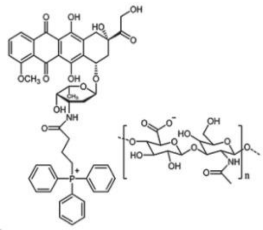	(Liu et al., 2018)

CDDO-Me	NF-κB pathway	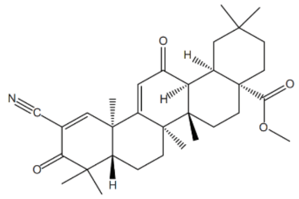	(Wang et al., 2014; Wang et al., 2021)
Triphenyl phosphonium derivatives of CDDO	Apoptosis	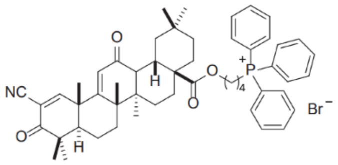	(Ju et al., 2021)
PAP-1 derivatives-PCARBTP	K+ ion channel	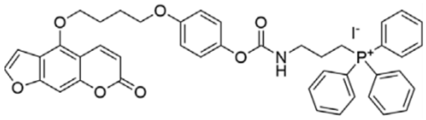	
PAP-1 derivatives-PAPOH	K+ ion channel	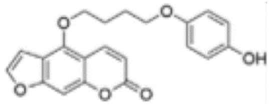	(Szabó et al., 2008)
PAP-1 derivatives-PAPTP	K+ ion channel	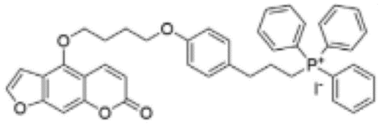	
Azathioprine	LC3 protein levels	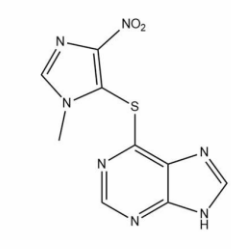	(Trybus et al., 2022)
SkQ1	free radicals	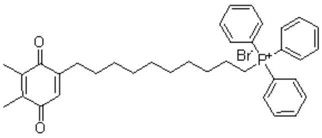	(Bazhin et al., 2016)
